# Identification of Two New Phenanthrenes from Dendrobii Herba and Their Cytotoxicity towards Human Hypopharynx Squamous Carcinoma Cell (FaDu)

**DOI:** 10.3390/molecules24122339

**Published:** 2019-06-25

**Authors:** Bomi Nam, Seung Mok Ryu, Dongho Lee, Chan-Hun Jung, Chang Hyun Jin, Jin-Baek Kim, Ik-Soo Lee, Ah-Reum Han

**Affiliations:** 1 Advanced Radiation Technology Institute, Korea Atomic Energy Research Institute, Jeongeup-si, Jeollabuk-do 56212, Korea; bomi1201@kaeri.re.kr (B.N.); chjin@kaeri.re.kr (C.H.J.); jbkim74@kaeri.re.kr (J.-B.K.); 2 College of Pharmacy, Chonnam National University, Gwangju 61186, Korea; 3 Department of Biosystems and Biotechnology, Korea University, Seoul 02841, Korea; smryu@kiom.re.kr (S.M.R.); dongholee@korea.ac.kr (D.L.); 4 Herbal Medicine Resources Research Center, Korea Institute of Oriental Medicine, Naju-si, Jeollanam-do 58245, Korea; 5 Department of Otolaryngology-Head & Neck Surgery, School of Medicine, Kyung Hee University, Seoul 02447, Korea; chjung@khu.ac.kr

**Keywords:** Dendrobii Herba, (1*R*,2*R*)-1,7-hydroxy-2,8-methoxy-2,3-dihydrophenanthrene-4(1*H*)-one, 2,7-dihydroxy-phenanthrene-1,4-dione, cytotoxicity, FaDu human hypopharynx squamous carcinoma cell

## Abstract

Two new phenanthrenes, (1*R*,2*R*)-1,7-hydroxy-2,8-methoxy-2,3-dihydrophenanthrene-4(1*H*)-one (**1**) and 2,7-dihydroxy-phenanthrene-1,4-dione (**2**), were isolated from the ethyl acetate-soluble fraction of Dendrobii Herba, together with seven known phenanthrenes (**3**–**9**), two bibenzyls (**10**–**12**), and a lignan (**13**). Structures of **1** and **2** were elucidated by analyzing one-dimensional (1D) and two-dimensional (2D)-NMR and High-resolution electrospray ionization mass spectra (HR-ESI-MS) data. The absolute configuration of compound **1** was confirmed by the circular dichroism (CD) spectroscopic method. In cytotoxicity assay using FaDu human hypopharynx squamous carcinoma cell line, compounds **3**–**6**, **8**, **10**, and **12** showed activities, with IC_50_ values that ranged from 2.55 to 17.70 μM.

## 1. Introduction

Dendrobii Herba is a herbal medicine that uses stems of *Dendrobium* species (Orchidacea), such as *D*. *nobile*, *D*. *chrysanthum*, *D*. *officinale*, *D*. *loddigessi*, *D*. *fimbriatum* var. *oculatum*, *D*. *moniliforme*, or *D*. *candidum* [1,2]. It has been traditionally used to treat lower fever, dryness of throat, gastrodynia due to stomach problem, blurred vision, and atrophy of the tendon and bone due to kidney problems in East Asia [3,4,5]. Previous phytochemical studies on *Dendrobium* species have reported the isolation of various types of compounds, specifically phenanthrenes as major components [6,7,8,9,10,11,12,13,14]. Phenanthrenes have been reported to have anti-inflammatory [6,7,8], antifibrotic [9], anti-cancer [10,11,12], and antibacterial activities [13]. Bibenzyl compounds, including stilbenes, are also abundant in *Dendrobium* species [14,15,16,17,18,19] with diverse activities, such as antioxidant [14], anti-inflammatory [14], anti-migratory [15], retinal neoangiogenesis inhibitory [16], and antimutagenic [17,18] activities.

Head and neck cancer is a group of cancers that primarily originate in the lips, mouth, nasal cavity, sinuses, and larynx. Head and neck squamous cell carcinoma (HNSCC) accounts for most of the cancers of the head. It arises from the mucosal surface of this part [20]. The most common risk factors that are associated with head and neck cancer are alcohol and tobacco [21]. Around the world, over 550,000 cases of HNSCC and around 300,000 deaths have been annually reported [21]. Although clinical trials, including surgery, radiation therapy, and chemotherapy, have been conducted, five-year survival rate of HNSCC patients has not improved over the past few decades [20,21]. Natural products with apoptotic mechanism in HNSCC have been reported as part of the effort to develop chemotherapeutic agents for HNSCC [22,23].

**Figure 1:**
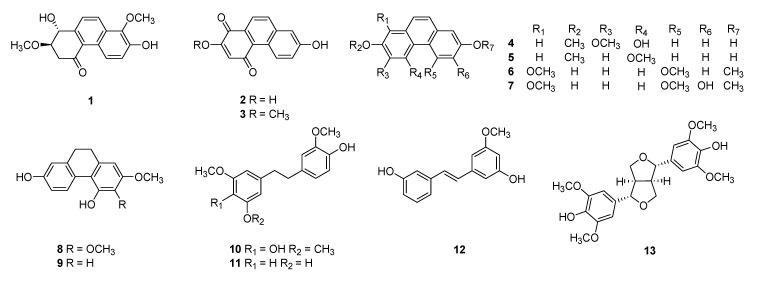
Chemical structures of compounds isolated from the ethyl acetate-soluble fraction of Dendrobii Herba.

During our screening procedure to find new bioactive compounds from plant sources, the ethyl acetate-soluble fraction of Dendrobii Herba exhibited considerable cytotoxicity against the FaDu human hypopharynx squamous carcinoma cell line, with an IC_50_ value of 13.16 μg/mL. Therefore, it was subjected to detailed phytochemical investigation, affording 13 compounds, including two new phenanthrenes **1** and **2** (Figure 1). Herein, we describe the structural elucidation of **1** and **2** and the results of biological evaluation for compounds **1**–**13**.

## 2. Results and Discussion

### 2.1. Structure Elucidation of Compounds ***1*** and ***2***

**Figure 2:**
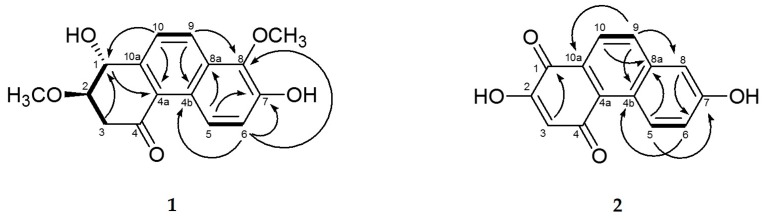
Key ^1^H-^1^H COSY (▬) and ^1^H-^13^C-HMBC-(→) correlations of **1** and **2**.

**Figure 3:**
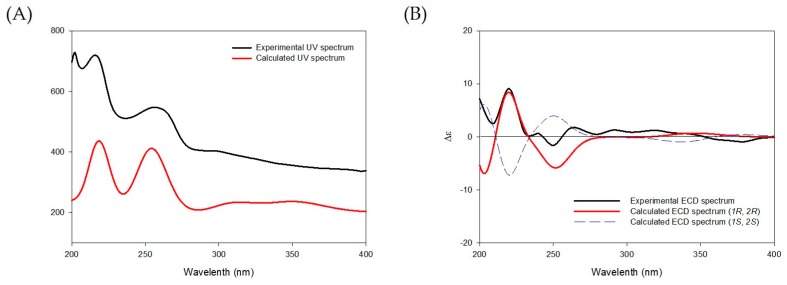
(**A**) Experimental and calculated UV spectra of **1** and (**B**) Experimental ECD spectra of **1** and the calculated ECD spectra of (1*R*,2*R*)-**1** and (1*S*,2*S*)-**1**.

Compound **1** was obtained as a brown solid with a molecular ion peak at *m*/*z* 311.0891 [M + Na]^+^ in high resolution electrospray ionization mass spectrum corresponding to an elemental formula of C_16_H_16_O_5_Na. In the ^1^H-NMR spectrum of **1**, two sets of *ortho*-coupled aromatic proton signals at δ_H_ 8.99 (1H, d, *J* = 9.0 Hz, H-5), 8.32 (1H, d, *J* = 8.8 Hz, H-9), 7.73 (1H, d, *J* = 8.8 Hz, H-10), and 7.24 (1H, d, *J* = 9.0 Hz, H-6) showed the two fused benzene ring system (Table 1). Thus, it was supported by ^1^H-^1^H COSY NMR correlations of H-5/H-6 and H-9/H-10 and ^1^H-^13^C-HMBC-NMR correlations of H-5/C-7, C-8a, H-6/C-4b, C-7, C-8, H-9/C-4b, C-8, and H-10/C-8a (Figure 2). The ^1^H and ^13^C-NMR spectra of **1** displayed signals for two oxygenated methine groups at δ_H_ 4.88 (1H, d, *J* = 8.5 Hz, H-1)/δ_C_ 70.9 (C-1) and 3.79 (1H, m, H-2)/80.5 (C-2), a methylene group at δ_H_ 3.22 (1H, dd, *J* = 16.2, 3.8 Hz, H-3α), and 2.74 (1H, dd, *J* = 16.2, 8.5 Hz, H-3β)/δ_C_ 42.2 (C-3), and a carbonyl group at δ_C_ 198.7 (C-4), representing a cyclohexanone ring that was linked to C-4a and C-10a in the naphthalene system by ^1^H-^13^C-HMBC-NMR correlations of H-1/C-2, C-4a, C-10a, H-3/C-1, C-2, C4, H-9/C-10a, and H-10/C-4a. Positions of two methoxy groups were assigned at C-2 and C-8, respectively, by ^1^H-^13^C-HMBC-NMR correlations of OCH_3_/C-2 and OCH_3_/C-8. In addition, when comparing ^1^H and ^13^C-NMR spectra of **1** with those of heliophenanthrene [24] indicated that the structure of **1** was similar to that of heliophenanthrene, except for the difference in signals of the aromatic ring system and functional groups. The *trans* stereochemistry between H-1 and H-2 was deduced by their large coupling constant (*J* = 8.5 Hz). The absolute configuration of **1** at C-1 and C-2 was assigned by comparing its experimental ECD spectrum to the calculated ECD spectra of two enantiomers (1*R*,2*R*)-**1** and (1*S*,2*S*)-**1** and determined as (1*R*,2*R*) due to the similarity of ECD spectrum of **1** with that of (1*R*,2*R*)-**1** (Figure 3). Therefore, the structure of compound **1** was elucidated as (1*R*,2*R*)-1,7-hydroxy-2,8-methoxy-2,3-dihydrophenanthrene-4(1*H*)-one.

Compound **2** was obtained as a brown solid with a molecular ion peak at *m*/*z* 239.0346 [M − H]^−^ in high resolution electrospray ionization mass spectrum, which is consistent with an elemental formula of C_14_H_7_O_4_. The ^1^H spectrum of **2** displayed signals for a 1,2,4-trisubstituted aromatic ring system at δ_H_ 9.41 (1H, d, *J* = 9.3 Hz, H-5), 7.26 (1H, dd, *J* = 9.3, 2.0 Hz, H-7) and 7.14 (1H, d, *J* = 2.0, H-8), *ortho*-coupled aromatic protons at δ_H_ 7.94 (1H, d, *J* = 8.8 Hz, H-9), and 8.03 (1H, d, *J* = 8.8 Hz, H-10), and an aromatic proton singlet at δ_H_ 6.18 (1H, s, H-2). The ^13^C spectrum of **2** showed signals for two conjugated carbonyl carbons at δ_C_ 180.9 (C-1) and 188.8 (C-4) (Table 1). These NMR data suggest that **2** has a phenathrenedione structure. This was supported by the ^1^H-^13^C-HMBC-NMR correlations of H-3/C-1, H-5/C-7, C-8a, H-6/C-4b, H-8/C-6, H-9/C-4b, C-8, C-10a, and H-10/C-8a (Figure 2). The few three bond correlations observed in the ^1^H-^13^C-HMBC-NMR spectrum of **2** did not allow for unambiguous assignment of ^13^C resonance at C-2 or C-3. However, further comparison of ^13^C-NMR data of two carbonyl groups with published values in 1,4-phenanthrenedione structure [14,25,26] confirmed that two carbonyl carbon signals at δ_C_ 180.9 and δ_C_ 188.8 could be assigned to C-1 and C-4, respectively. Accordingly, the position of a hydroxyl group in the 1,4-benzoquinone moiety was determined as C-2 by three-bond correlation from the aromatic proton signal at δ_H_ 6.18 (1H, s, H-2) to the carbonyl carbon signal at δ_C_ 180.9 (C-1). Another hydroxyl group was attached at C-7, as evidenced by the ^1^H-^13^C-HMBC-NMR correlations of H-5/C-7. Furthermore, the ^1^H and ^13^C spectra (in DMSO-*d*_6_) of **2** showed almost identical signals to those of **3** [14], except for the absence of signals for a methoxy group at δ_H_ 3.86 (3H, s, 2-OCH_3_)/δ_C_ 57.0 (OCH_3_) in **3** (Supplementary Materials Table S1). Therefore, its structure was determined to be 2,7-dihydroxy-phenanthrene-1,4-dione.

The eleven known compounds were identified as densiflorol B (**3**) [14], 6,7-dimethoxy-phenanthrene-2,5-diol (**4**) [27], dehydroorchinol (**5**) [8], 1,5,7-trimethoxy-2-phenanthrenol (**6**) [8], denthyrsinin (**7**) [28], ephemeranthol A (**8**) [29], lusianthridin (**9**) [30], moscatilin (**10**) [18], gigantol (**11**) [12], 3-[(*1E*)-2-(3-Hydroxyphenyl)ethenyl]-5-methoxyphenol (**12**) [31], and (−)-syringaresinol (**13**) [19] by comparing their spectroscopic data with the published data (Supplementary Materials Figures S11–S32). Although the known compounds (**3**–**11** and **13**) have been isolated from *Dendrobium* species, the isolation of compound **12** from *Dendrobium* species has not been reported yet.

### 2.2. Biological Activity

The ethanol extract and solvent fractions of Dendrobii Herba primarily tested their cytotoxic activities on human pharynx squamous carcinoma (FaDu) cell line. The ethanol extract showed the activity with IC_50_ value of 16.57 μg/mL. It was successively partitioned with hexanes, ethyl acetate, and *n*-butanol, and these solvent fractions exhibited their cytotoxicities with IC_50_ values of 14.51, 13.16, and 13.67 μg/mL, respectively (Supplementary Materials Figure S33). The most active fraction, the ethyl acetate fraction, was subjected to detailed laboratory investigation in order to isolate the active compounds. All of the isolates were evaluated for their cytotoxicities to FaDu cell line and, of them, compounds **3**–**6**, **8**, **10**, and **12** exhibited cytotoxicities with IC_50_ values of 15.91, 11.40, 17.33, 17.70, 17.03, 2.55, and 17.14 μM, respectively (Supplementary Materials Figure S34). Cisplatin as a positive control showed an IC_50_ value of 1.18 μM. The structural differences influenced the potency of cytotoxicity. In broad outlines, the methylation of the free hydroxyl groups and the presence of additional oxygenated groups in both aromatic rings in each skeleton group, phenanthrene (**3**–**6**), 9,10-dihydrophenanthrene (**8**), or bibenzyl (**10**) were deduced to be more active. Of these active compounds, compound **10** showed the strongest cytotoxicity. Previous studies regarding the anticancer activities of **10** have reported that **10** can induce apoptosis of human pancreatic cancer cells though reactive oxygen species (ROS) and the c-Jun N-terminal kinase/stress-activated protein kinase (JNK/SAPK) pathway [32], apoptosis of human esophageal cancer cells by G2/M arrest and protein regulating mitosis [33], and the apoptosis of human colorectal cancer cells via JNK activation by tubulin depolymerization and DNA damage [34]. There are also reports on the regulation of tumor cell metastasis by **10**. For example, it has been reported that compound **10** can inhibit lung cancer cell migration and invasion through the suppression of ROS and focal adhesion kinase/protein kinase B (FAK/Akt) activation [35]. It can also inhibit breast cancer cell migration by inhibiting Akt/Twist [36]. In addition, compound **10** has an anti-angiogenesis effect on human umbilical vein endothelial cells via Extracellular signal-regulated kinases (ERK1/2), Akt, and endothelial nitric oxide synthase (eNOS) signaling pathways [37]. However, to the best of our knowledge, there has been no report of an anticancer mechanistic study of compound **10** in HNSCC, including FaDu cells. Thus, **10** could be beneficial for treating human pharynx squamous cancer. However, further studies are needed to determine its mechanism of action while using in vitro and in vivo models.

## 3. Materials and Methods

### 3.1. General Procedures

Optical rotations were measured on a JASCO P-2000 polarimeter (JASCO Co., Tokyo, Japan). Circular dichroism (CD) measurements were performed while using a JASCO J-810 CD-ORD spectropolarimeter (JASCO Co., Tokyo, Japan). One-dimensional (1D) and two-dimensional (2D)-NMR experiments were performed on a JNM-ECA 500 MHz NMR instrument (JEOL Ltd., Tokyo, Japan) with tetramethylsilane (TMS) as the internal standard. High-resolution electrospray ionization mass spectra (HR-ESI-MS) were recorded on a Waters SYNAPT G2 mass spectrometer (Waters, Milford, MA, USA). Silica gel (70–230 mesh, Merck, Darmstadt, Germany), RP-18 (YMC gel ODS-A, 12 nm, S-75 μm, YMC Co., Tokyo, Japan), and Sephadex LH-20 (GE Healthcare Bio-Sciences, Uppsala, Sweden) were used for column chromatography (CC). Thin-layer chromatographic (TLC) analysis was performed on Kieselgel 60 F_254_ (Merck, Darmstadt, Germany) and Kieselgel 60 RP-18-F_254S_ (Merck, Darmstadt, Germany), with visualization under UV light (254 and 365 nm) and 10% (*v*/*v*) sulfuric acid spray, followed by heating at 180 °C for 2 min. YMC-Pack Pro C18 column (5 μm, 250 mm × 20 mm i.d., YMC Co., Tokyo, Japan) was used for preparative high performance liquid chromatography (HPLC) that was conducted on a Gilson Preparative HPLC system (Gilson Inc., Middleton, WI, USA). Medium pressure liquid chromatography (MPLC) was performed on a CombiFlash Rf200 system (Teledyne ISCO, Lincoln, NE, USA) with Redi*Sep* Rf Normal Phase Silica columns. Analytical HPLC-DAD was carried out on an Agilent 1200 series system (Agilent Technologies Co., Santa Clara, CA, USA) that was equipped with a YMC-Triart C18 column (5 μm, 250 mm × 4.6 mm, YMC Co., Tokyo, Japan).

### 3.2. Plant Material

Dendrobii Herba stems (CK PHARM Co., Ltd., Seoul, Korea) were purchased from the Jewondang herb shop in Jeongup-si, Jeollabuk-do, Korea. Voucher specimens (accession no. TM007) were deposited at the Advanced Radiation Technology Institute, Korea Atomic Energy Research Institute. HPLC-DAD analysis was performed on its ethanol extract and solvent fractions to confirm the quality of this plant material (Supplementary Materials Figure S35).

### 3.3. Extraction and Isolation

Dried stems (5 kg) of Dendrobii Herba were extracted with 95% EtOH (5 × 14 L) overnight at room temperature. The solvent was evaporated in vacuo to afford a 95% EtOH extract (122 g), which was then suspended in distilled water (1 L) and partitioned with hexanes (3 × 1 L), ethyl acetate (5 × 1 L), and *n*-butanol (2 × 1 L) sequentially. The EtOAc-soluble fraction (35 g) was then subjected to silica gel column chromatography (CC) while using a radient solvent system of CHCl_3_-MeOH (1:0 to 0:1, *v/v*) to afford 16 fractions (F01–F16). Fraction F03 (0.7 g) was subjected to reverse-phase CC with a solvent system of MeOH-H_2_O (1:2 to 1:0, *v*/*v*), affording 15 subfractions (F0301–F0315). Subfraction F0311 (250 mg) was purified by MPLC (hexane-EtOAc, 85:15, 15 mL/min) to yield **8** (6 mg). Subfraction F0312 (115 mg) was chromatographed on a Sephadex LH-20 column while using 100% MeOH to give **7** (56 mg) and **10** (14 mg). Subfraction F0314 (80 mg) was separated by MPLC (heaxen-EtOAc, 9:1 to 85:15, 15 mL/min), affording two subfractions (F031401 and F031402). Purified F031401 was subjected to Sephadex LH-20 CC while using CHCl_3_-MeOH (1:1, *v*/*v*) to obtain **6** (1 mg) and **5** (2.5 mg). Fraction F 04 (6 g) was subjected to silica gel CC with a solvent system of CHCl_3_-MeOH (9.5:0.5 to 1:1, *v*/*v)*, which afforded ten subfractions (F0401–F0412). Subfraction F0404 (0.9 g) was subjected to reverse-phase CC with solvent system of MeOH-H_2_O (1:1 to 1:0, *v/v*), affording 20 subfractions (F040401–F040420). Subfraction F040412 was chromatographed on a Sephadex LH-20 column while using 100% MeOH to give **4** (3 mg). Fraction F07 (3 g) was subjected to silica gel CC with solvent system of heaxen-EtOAc (9:1 to 85:15, *v*/*v*), affording ten subfractions (F0701–F0710). Subfraction F0703 (126 mg) was subjected to Sephadex LH-20 CC while using 100% MeOH to give five subfractions (F070301–F070305). Subfraction F070305 (50 mg) was chromatographed on a Sepadex LH-20 column using 100% MeOH to give four subfractions (F07030501–F07030504). The fourth fraction (20 mg) was purified by reverse-phase CC with a gradient solvent system of MeOH-H_2_O (1:1 to 4:1, *v*/*v*) to yield **9** (10 mg). Subfraction F0704 (270 mg) was subjected to reverse-phase CC while using MeOH-H_2_O (1:1 to 1:0, *v*/*v*), producing 20 subfractions (F070401–F070420). Subfraction F070414 (14 mg) was chromatographed on a Sephadex LH-20 column while using CHCl_3_-MeOH (1:1, *v*/*v*) to give four fractions (F07041401–F07041404). F07041402 and F07041403 were purified by preparative HPLC (MeOH-H_2_O, 8:2, 3 mL/min) to afford **11** (2 mg, *t*_R_ 22.5 min) and **3** (1.6 mg, *t*_R_ 29.8 min). **2** (2 mg, *t_R_* 32.5 min) was obtained by preparative HPLC with (MeOH-H_2_O, 7.5:2.5, 3 mL/min). Subfraction F070416 (23 mg) was separated by Sephadex LH-20 CC using 50% MeOH in CHCl_3_ to give four subfractions (F07041601–F07041604). F07041601 (3.8 mg) was purified by preparative HPLC (MeOH- H_2_O, 7:3, 3 mL/min) to afford **12** (2.8 mg, *t*_R_ 40.3 min). F07041602 (3 mg) was purified by preparative HPLC (MeOH-H_2_O, 7:3, 3 mL/min) to obtain **13** (1.5 mg, *t*_R_ 36 min). Subfraction F0707 (120 mg) was subjected to reverse-phase CC using MeOH-H_2_O (1:1 to 1:0, *v*/*v*), affording 12 subfractions (F070701–F070712). The second subfraction was then purified by Sephadex LH-20 CC while using 100% MeOH to afford **1** (10 mg).

(1*R*,2*R*)-1,7-hydroxy-2,8-methoxy-2,3-dihydrophenanthrene-4(1*H*)-one (**1**): Brown solid. ${\left[\alpha \right]}_{D}^{\mathrm{20}}$ − 8.83° (*c* 0.03, MeOH). ^1^H (500 MHz) and ^13^C (125 MHz) data (CD_3_OD), see Table 1. HR-ESI-MS (positive ions) *m*/*z* 311.0891 [M + Na]^+^ (calculated for C_16_H_16_O_5_Na, 311.0895).

2,7-dihydroxy-phenanthrene-1,4-dione (**2**): Brown solid. ^1^H (500 MHz) and ^13^C (125 MHz) data (CD_3_OD), see Table 1. HR-ESI-MS (negative ions) *m/z* 239.0399 [M − H]^−^ (calculated for C_14_H_7_O_4_, 239.0344).

### 3.4. Computational Method

Conformer distributions, optimizations, and ECD calculations of compound **1** were carried out, as described previously [32]. Briefly, a conformer distribution was performed Spartan’14 software (Wave-function, Inc., Irvine, CA, USA) while using an MMFF force field. These conformers were optimized at DFT [B3LYP functional/6-31+G(d,p) basis set] level. ECD calculations were carried out at TDDFT (CAM-B3LYP/SVP basis set) level with a CPCM solvent model in MeCN using Gaussian 09 software (Gaussian, Inc., Wallingford, CT, USA). The calculated ECD spectra were simulated using SpecDis 1.64 software (University of Wuerzburg, Wuerzburg, Germany) with a half bandwidth of 0.3 eV. ECD curves of these conformers were weighted by the Boltzmann distribution.

### 3.5. HPLC Analysis

The ethanol extract and solvent fractions of Dendrobii Herba were accurately weighed and dissolved in MeOH at 2 mg/mL. The sample solution was filtered through a syringe filter (0.45 μm) for HPLC analysis. The standards were accurately weighed and dissolved in MeOH at 1.0 mg/mL for co-injection with each sample. Analysis of the chemical composition of the sample was conducted while using the Agilent 1200 series LC system with an YMC-Triart C18 column (5 μm, 250 mm × 4.6 mm, YMC Co.). Binary gradient elution with water (solvent A) and acetonitrile (solvent B) was performed, as follows: 0–5 min, 20% B; 5–45 min, 20–70% B; 45–46 min, 70–100% B; 46–56 min, 100% B; 56–57 min, and 100–20% B; 57–60 min, 20% B. The total flow rate was maintained at 1 mL/min and the injection volume was 10 μL. Chromatograms were acquired at 230, 254, and 280 nm by the DAD detector.

### 3.6. Cytotoxicity Assay

FaDu human pharynx squamous carcinoma cells were purchased from the Korean cell line bank (Seoul, Korea). All of the experiments were conducted with low-passage cell cultures (<passage 10). These cells were cultured in Minimum Essential Medium (MEM; Corning, Manassas, VA, USA) that was supplemented with 10% heat-inactivated FBS (Hyclone, Logan, UT, USA) in a humidified incubator with 5% CO_2_ at 37 °C. To determine viability of FaDu cells, the CCK-8 assay kit (Dojindo, Kumamoto, Japan) was used according to the manufacture’s protocols. Briefly, the FaDu cells were seeded into 96-well plates at a density of 0.2 × 10^5^ cells/mL and incubated at 37 °C for 24 h. After incubation, the cultured FaDu cells were treated with the indicated concentration of each compound (0.47−30 μM) and each extract (1.5625−100 μg/mL) for 72 h. Thereafter, 10 μL of CCK-8 reagent was added into cultured FaDu cells and then incubated for a further 4 h and absorbance was measured at 450 nm while using an SPARK^®^ multimode microplate reader (Tecan, Männedorf, Switzerland). Afterwards, 50% inhibitory concentration (IC_50_) was calculated from a dose-response analysis that was performed with GrahPad Prism software (GaraphPad Software, La Jolla, CA, USA).

## 4. Conclusions

Phytochemical study of Dendrobii Hereba resulted in isolation of two new phenanthrenes, (1*R*,2*R*)-1,7-hydroxy-2,8-methoxy-2,3-dihydrophenanthrene-4(1*H*)-one (**1**) and 2,7-dihydroxy-phenanthrene-1,4-dione (**2**). Of the 11 known compounds, compound **12** was isolated from *Dendrobium* species for the first time in this study. Compounds **3**–**6**, **8**, **10**, and **12** showed cytotoxicity to the FaDu cells, with moscatilin (**10**) exhibiting remarkable cytotoxic activity. Further mechanistic studies are needed to determine the anticancer action of **10** against head and neck cancers while using in vitro and in vivo models.

## Figures and Tables

**Table 1:** ^1^H-NMR (500 MHz) and ^13^C-NMR (125 MHz) spectral data (CD_3_OD, δ in ppm) of **1** and **2** isolated from Dendrobii Herba.

Position	1		2	
	δ_H_	δ_C_	δ_H_	δ_C_
1	4.88 (4H, d, *J* = 8.5 Hz)	70.9		180.9
2	3.79 (1H, m)	80.5		159.0
3			6.18 (1H, s)	110.6
3α	3.22 (1H, dd, *J* = 16.2, 3.8 Hz)	42.2		
3β	2.74 (1H, dd, *J* = 16.2, 8.5 Hz)			
4		198.7		188.8
5	8.99 (1H, d, *J* = 9.0 Hz)	123.3	9.41 (1H, d, *J* = 9.3 Hz)	129.8
6	7.24 (1H, d, *J* = 9.0 Hz)	121.1	7.26 (1H, dd, *J* = 9.3, 2.0 Hz)	122.5
7		146.5		158.7
8		140.1	7.14 (1H, d, *J* = 2.0 Hz)	109.6
9	8.32 (1H, d, *J* = 8.8 Hz)	127.3	7.94 (1H, d, *J* = 8.8 Hz)	131.8
10	7.73 (1H, d, *J* = 8.8 Hz)	125.8	8.03 (1H, d, *J* = 8.8 Hz)	121.6
4a		126.0		127.4
4b		129.6		123.8
8a		125.4		139.8
10a		142.4		128.3
2-OCH_3_	3.47 (3H, d, *J* = 1.5 Hz)	56.2		
8-OCH_3_	3.91 (3H, d, *J* = 1.5 Hz)	60.2		

## Supplementary Materials

The supplementary materials are available online.
