# Supplementary material for: Identification of Two New Phenanthrenes from Dendrobii Herba and Their Cytotoxicity towards Human Hypopharynx Squamous Carcinoma Cell (FaDu)
Source: Molecules. 2019 Jun 25;24(12):2339. doi: 10.3390/molecules24122339 (PMC6631096; doi:10.3390/molecules24122339)
Supplement: Supplementary file 1 [file molecules-24-02339-s001.pdf]

Supplementary Materials: **Identification of two new phenanthrenes from Dendrobii Herba and their cytotoxicity towards human hypopharynx squamous carcinoma cell (FaDu)**

Bomi Nam, Seung Mok Ryu, Dongho Lee, Chan-Hun Jung, Chang Hyun Jin, Jin-Baek Kim, Ik-Soo Lee* and Ah-Reum Han*

Table **S1**. ^{1}H-NMR (500 MHz) and ^{13}C-NMR (125 MHz) spectral data of **2** and **3** in DMSO-d_6 (δ in ppm)

Position	**2**		**3**	
	δ_H	δ_C	δ_H	δ_C
1		179.9		180.8
2		158.5		158.9
3	6.22 (1H, s)	110.8	6.29 (1H, s)	117.8
4		188.8		189.0
5	9.24 (1H, d, J = 9.8 Hz)	129.4	9.36 (1H, d, J = 9.5 Hz)	128.8
6	7.19 (1H, dd, $J_{1,2}$ = 9.8, 1.8 Hz)	123.8	7.26 (1H, dd, $J_{1,2}$ = 9.5, 2.8 Hz)	123.1
7		157.0		158.3
8	7.02 (1H, brs)	109.9	7.25 (1H, d, J = 2.8 Hz)	110.3
9	7.87 (2H, d, J = 8.3 Hz)	131.2	8.08 (1H, d, J = 8.3 Hz)	132.9
10		128.5	7.97 (1H, d, J = 8.3 Hz)	130.2
4a		127.0		127.4
4b		122.4		123.9
8a		141.2		139.5
10a		121.6		122.4
2-OCH$_3$			3.86 (3H, s)	57.0

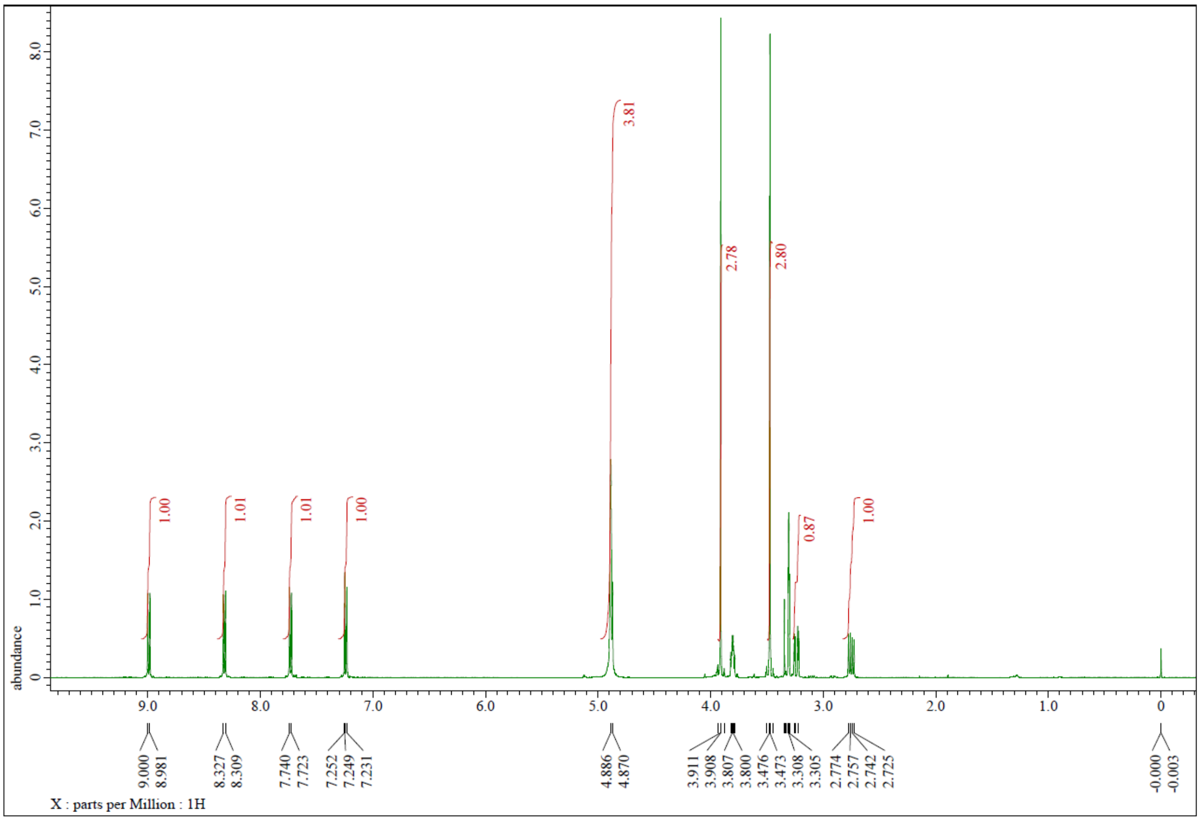

Figure S1. ^{1}H-NMR (500 MHz, CD_3OD) spectrum of compound **1**.

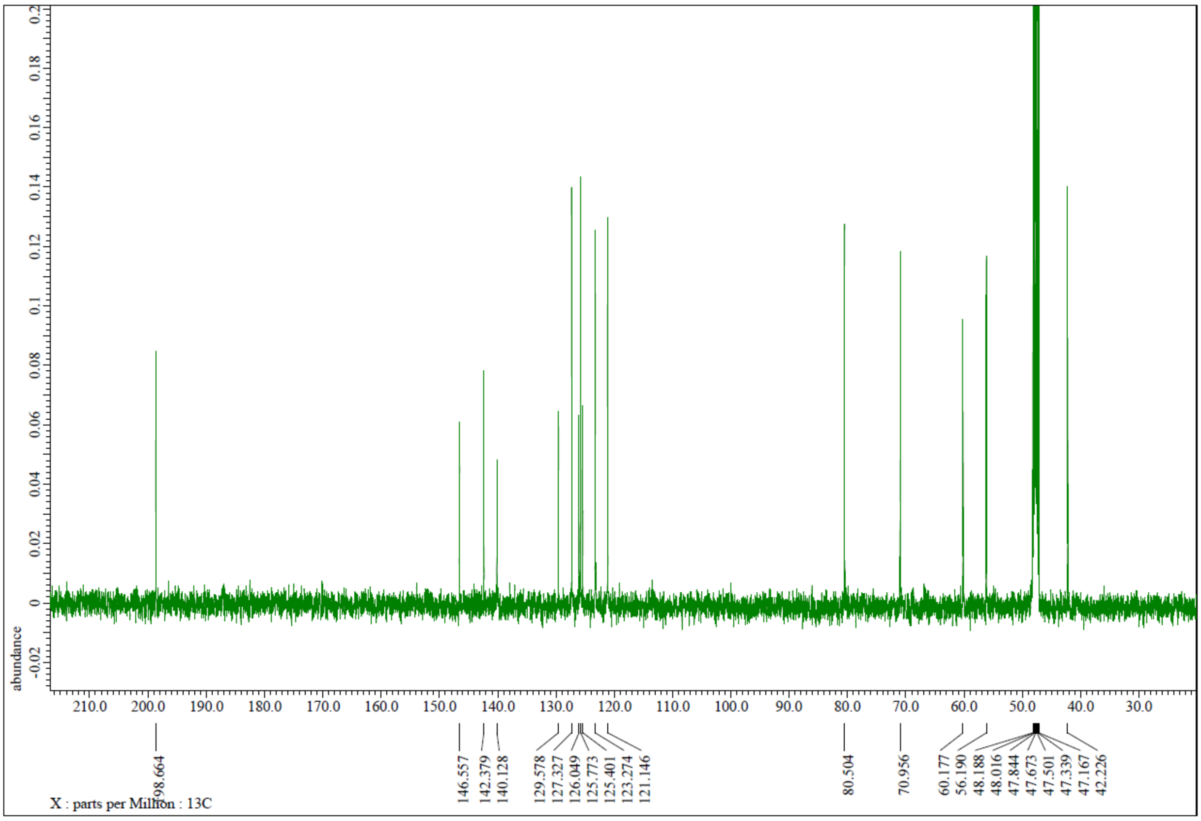

Figure S2. ^{13}C-NMR (125 MHz, CD_3OD) spectrum of compound **1**.

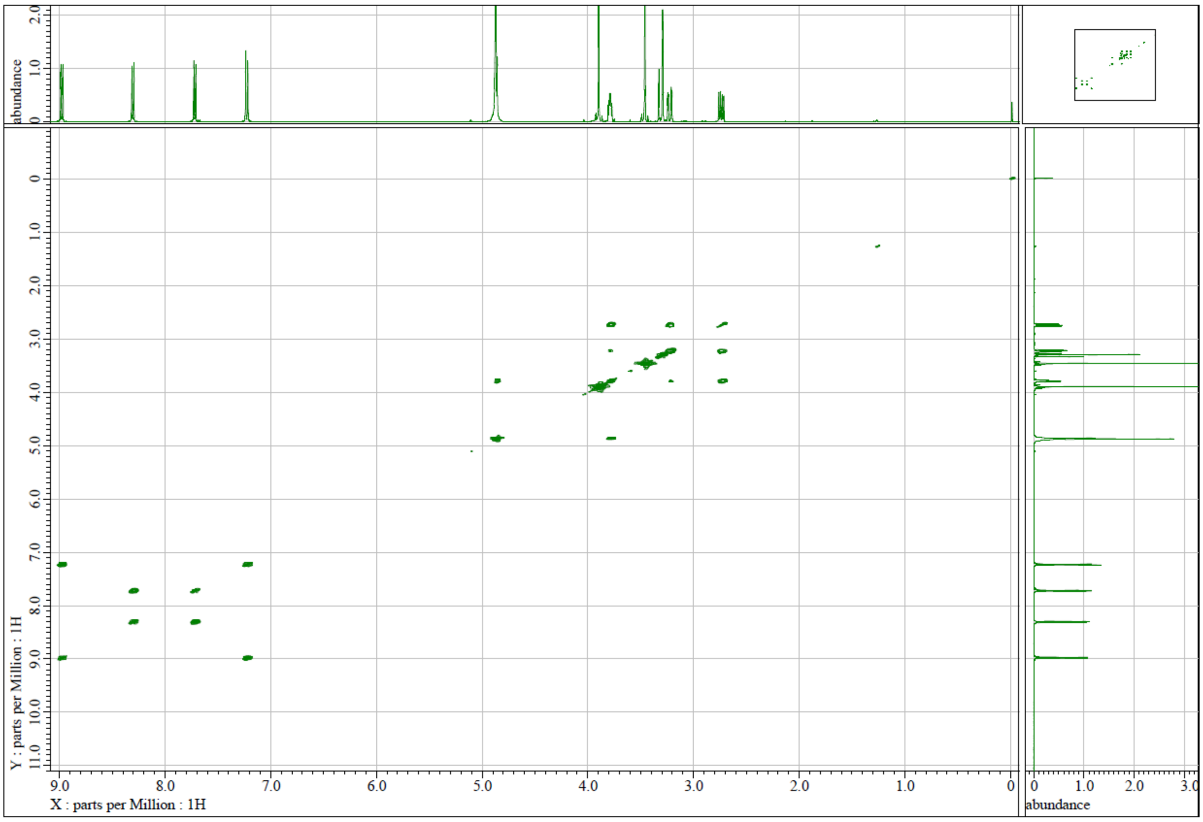

Figure S3. ^{1}H-^{1}H COSY NMR spectrum of compound **1**.

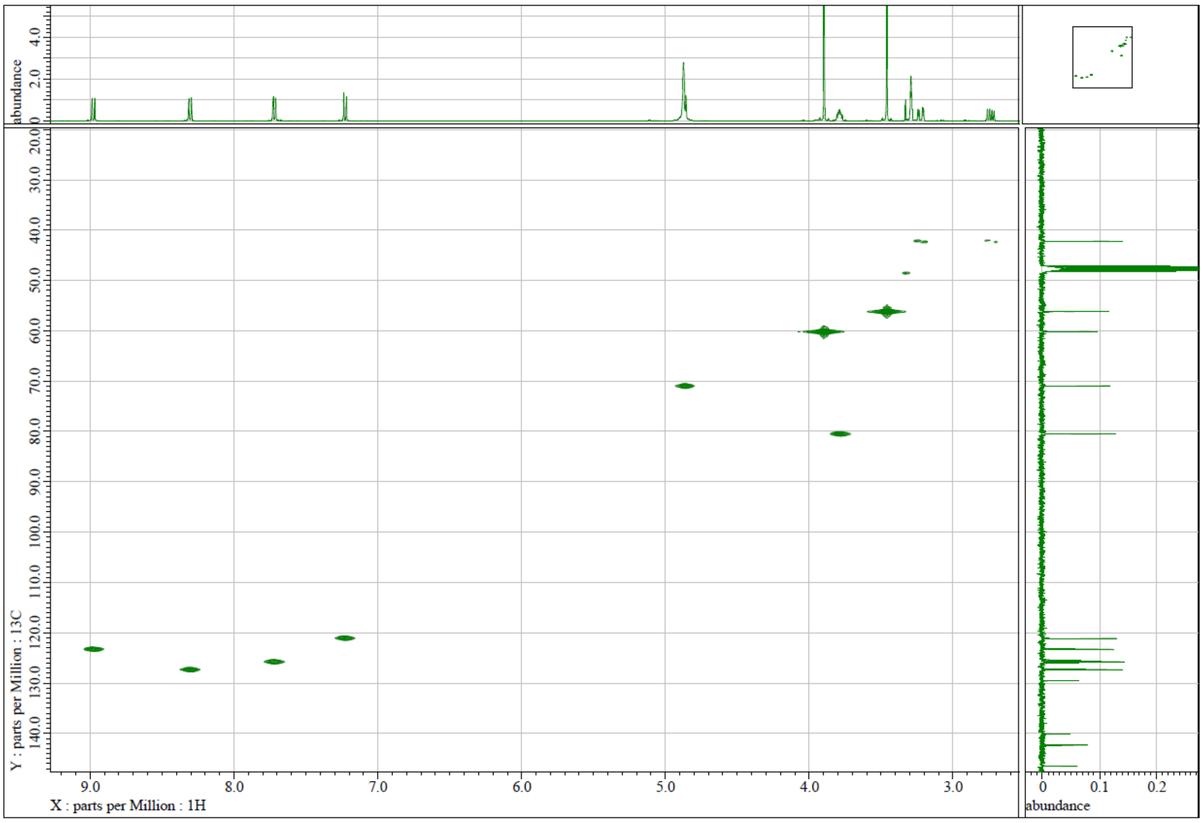

Figure S4. ^{1}H-^{13}C HMQC NMR spectrum of compound **1**.

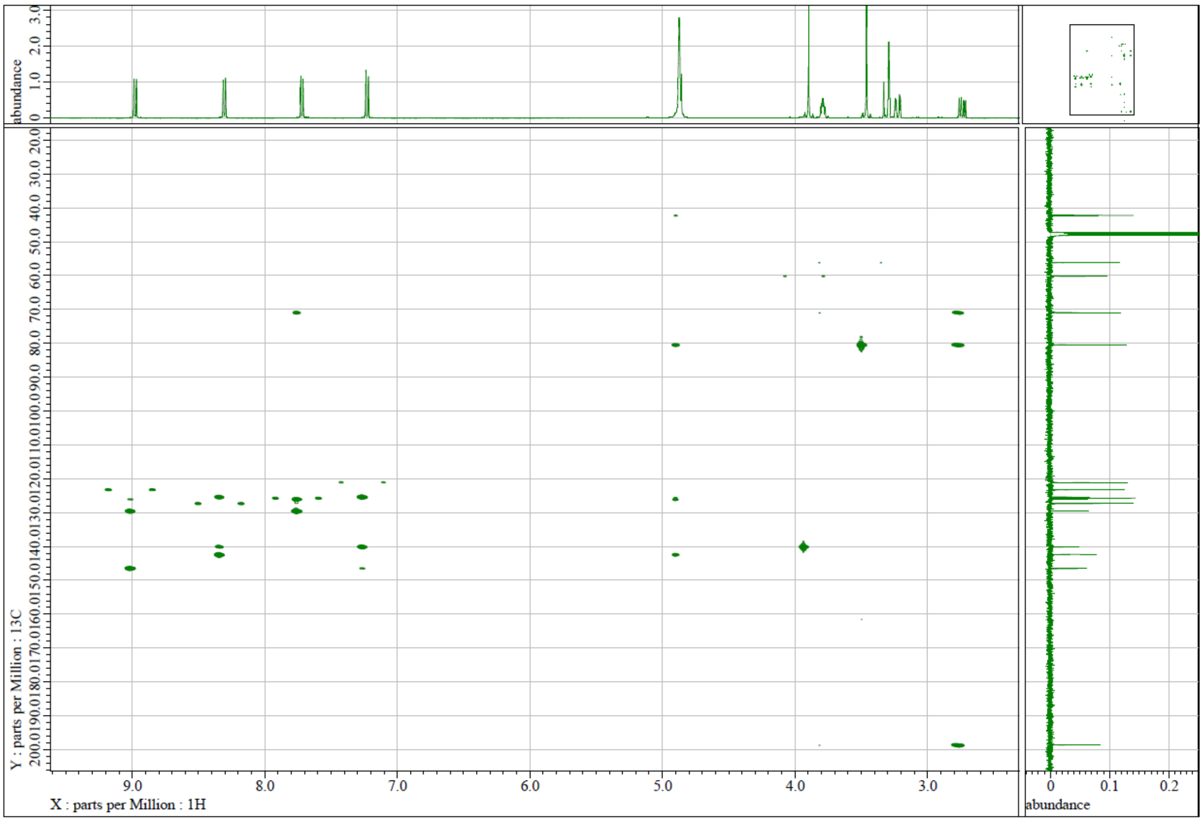

Figure S5. ^{1}H-^{13}C HMBC NMR spectrum of compound **1**.

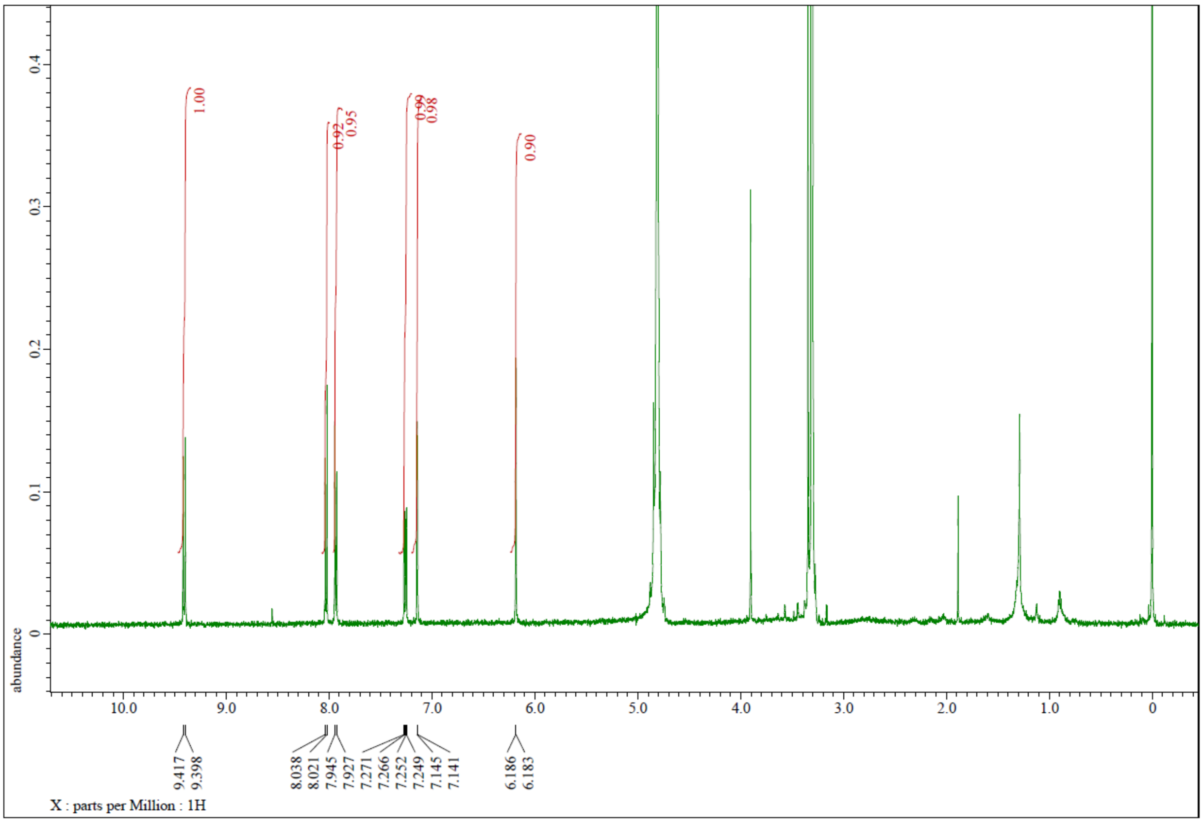

Figure S6. ^{1}H-NMR (500 MHz, CD_3OD) spectrum of compound **2**.

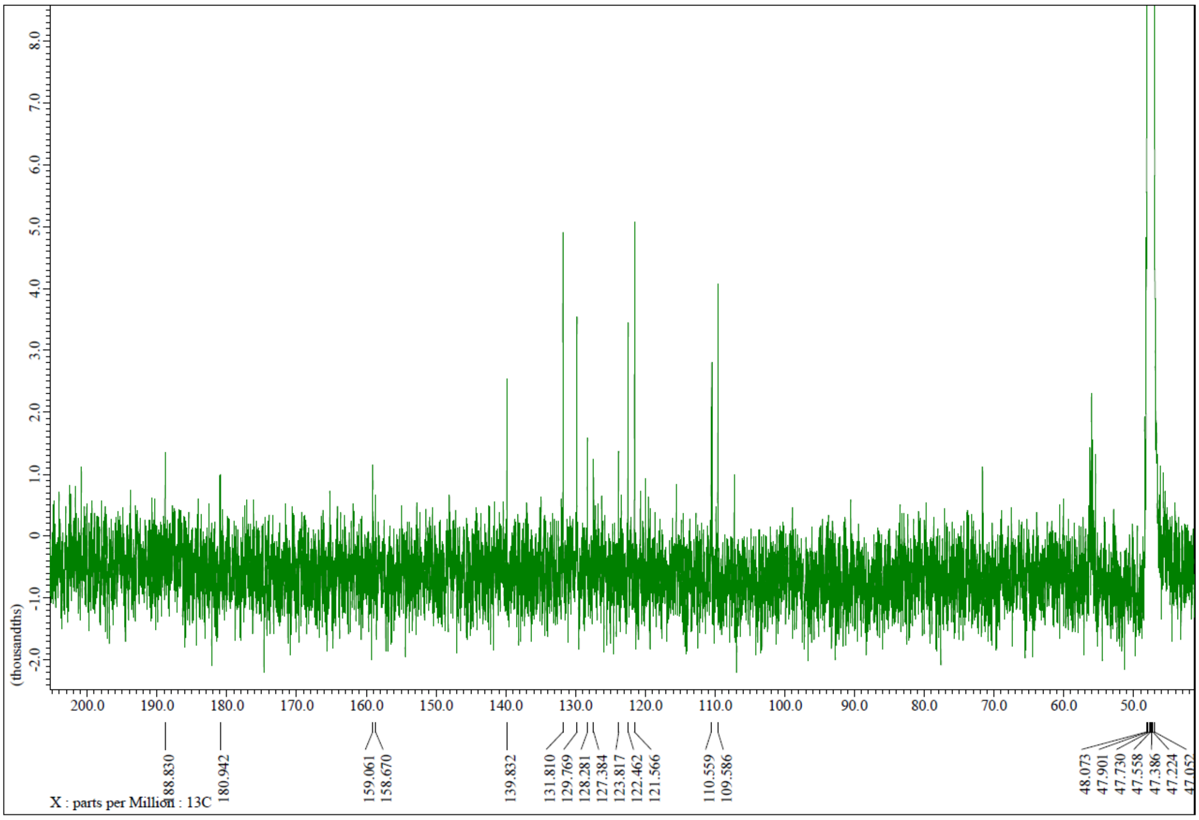

Figure S7. ^{13}C -NMR (125 MHz, CD_3OD) spectrum of compound **2**.

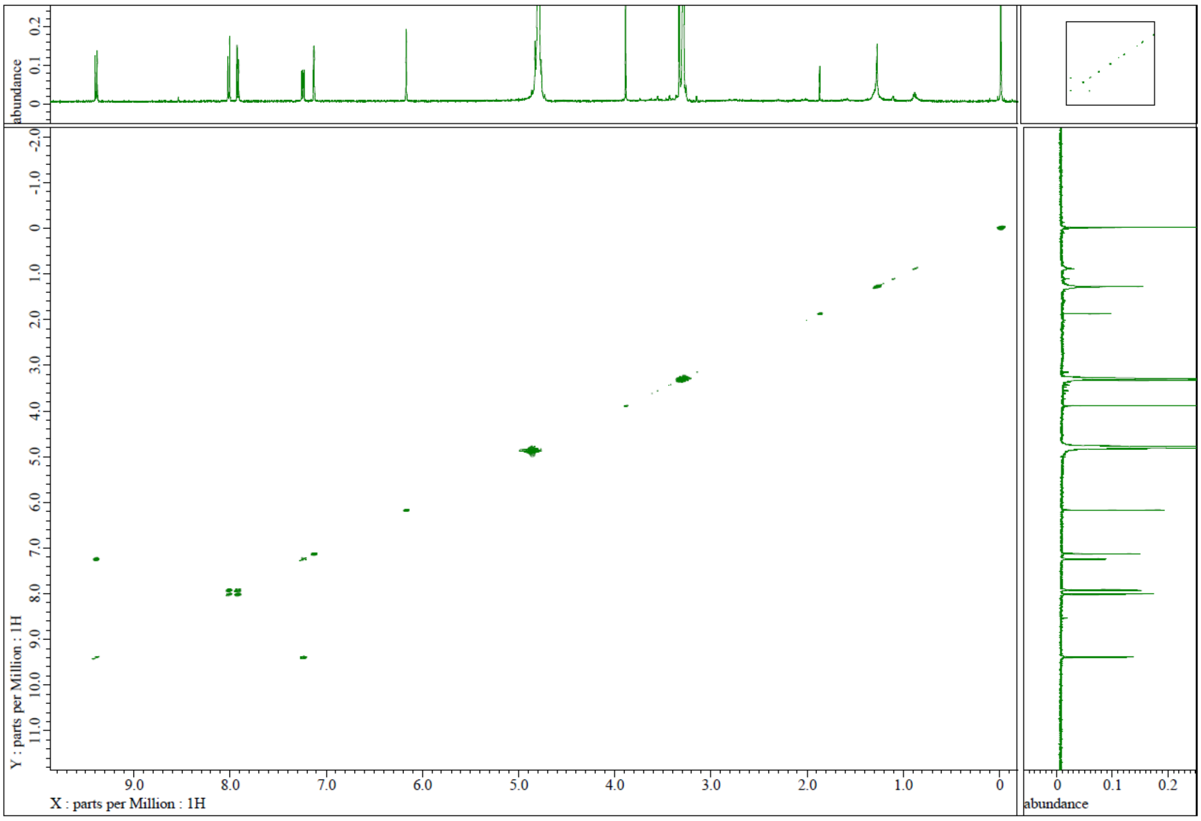

Figure S8. ^{1}H-^{1}H COSY NMR spectrum of compound **2**.

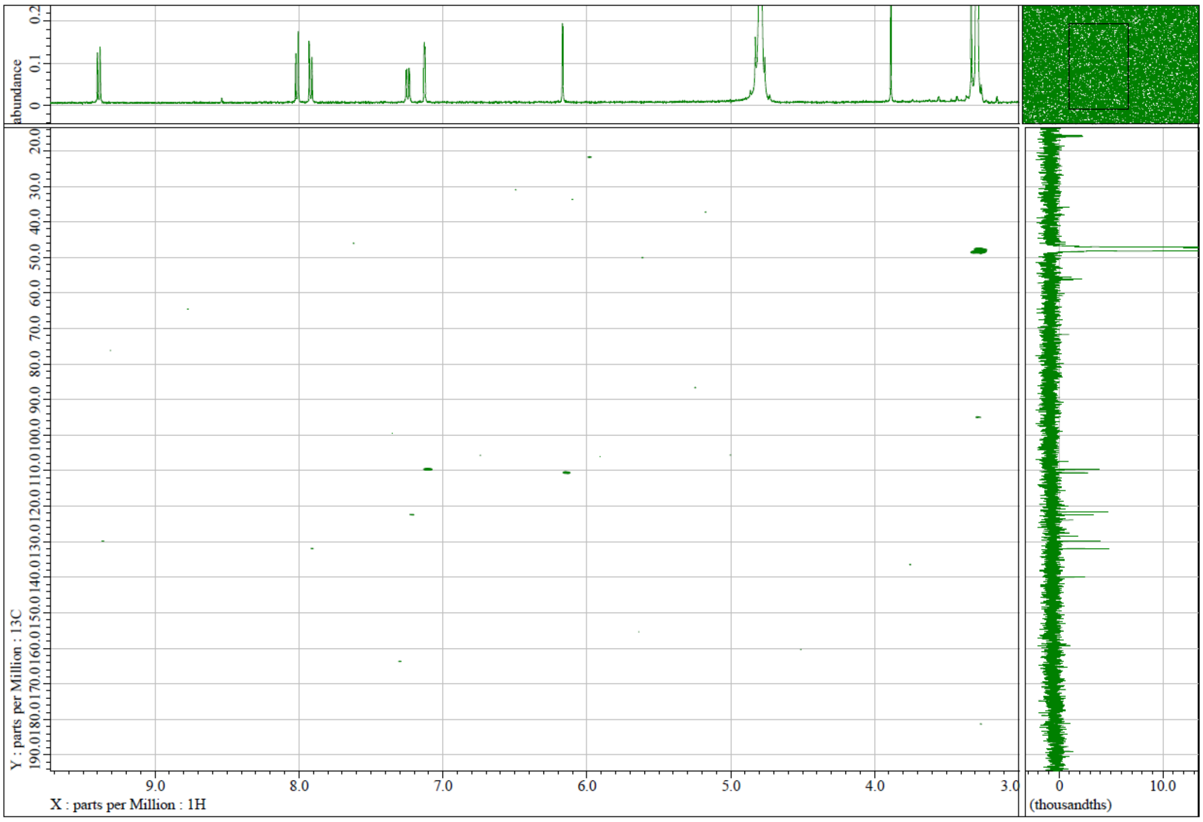

Figure S9. ^{1}H-^{13}C HMQC NMR spectrum of compound **2**.

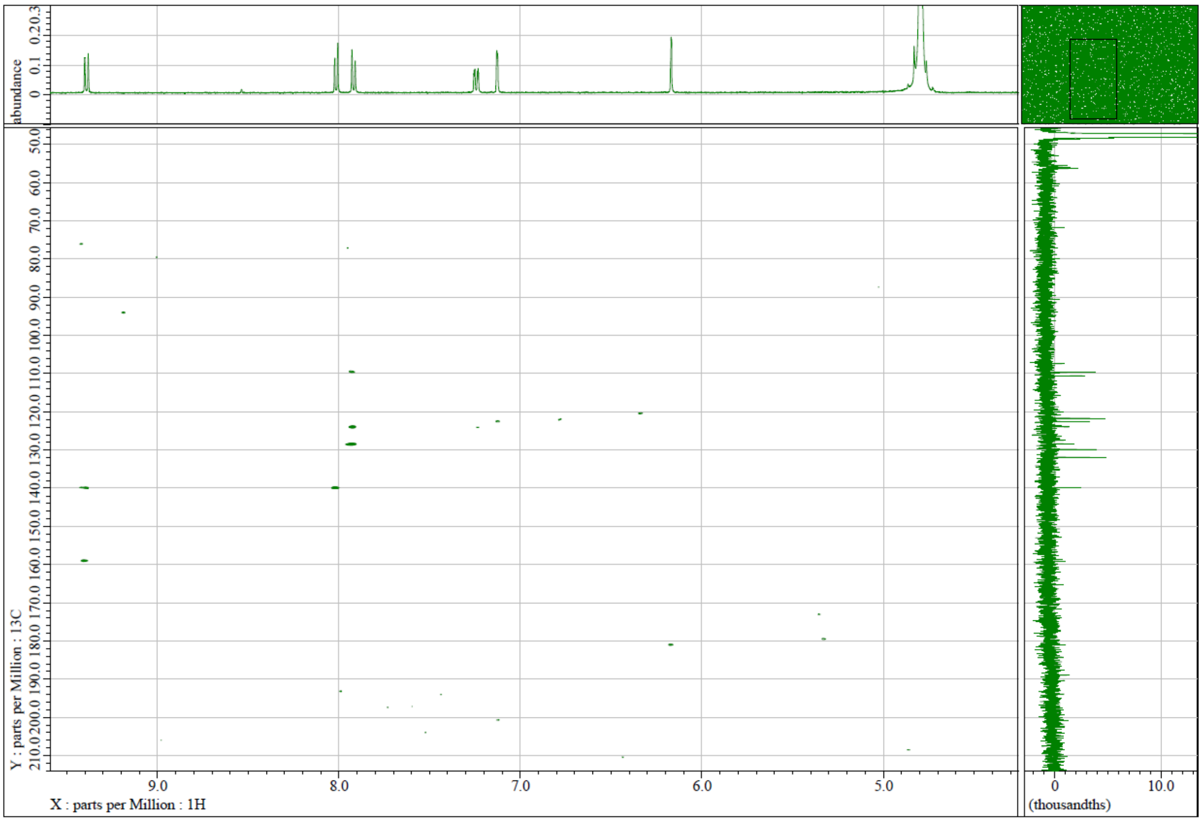

Figure S10. ^{1}H-^{13}C HMBC NMR spectrum of compound **2**.

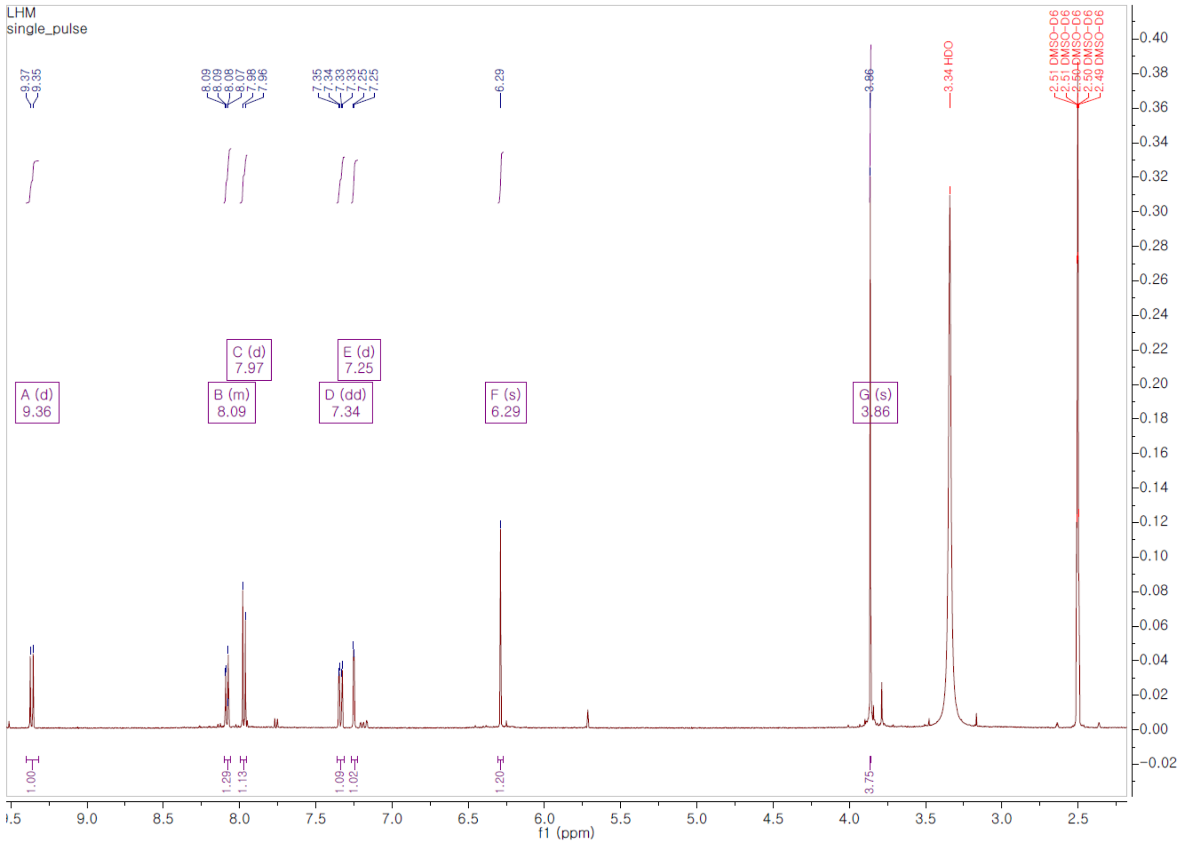

Figure S11. ^{1}H-NMR (500 MHz, DMSO-*d6*) spectrum of compound **3**.

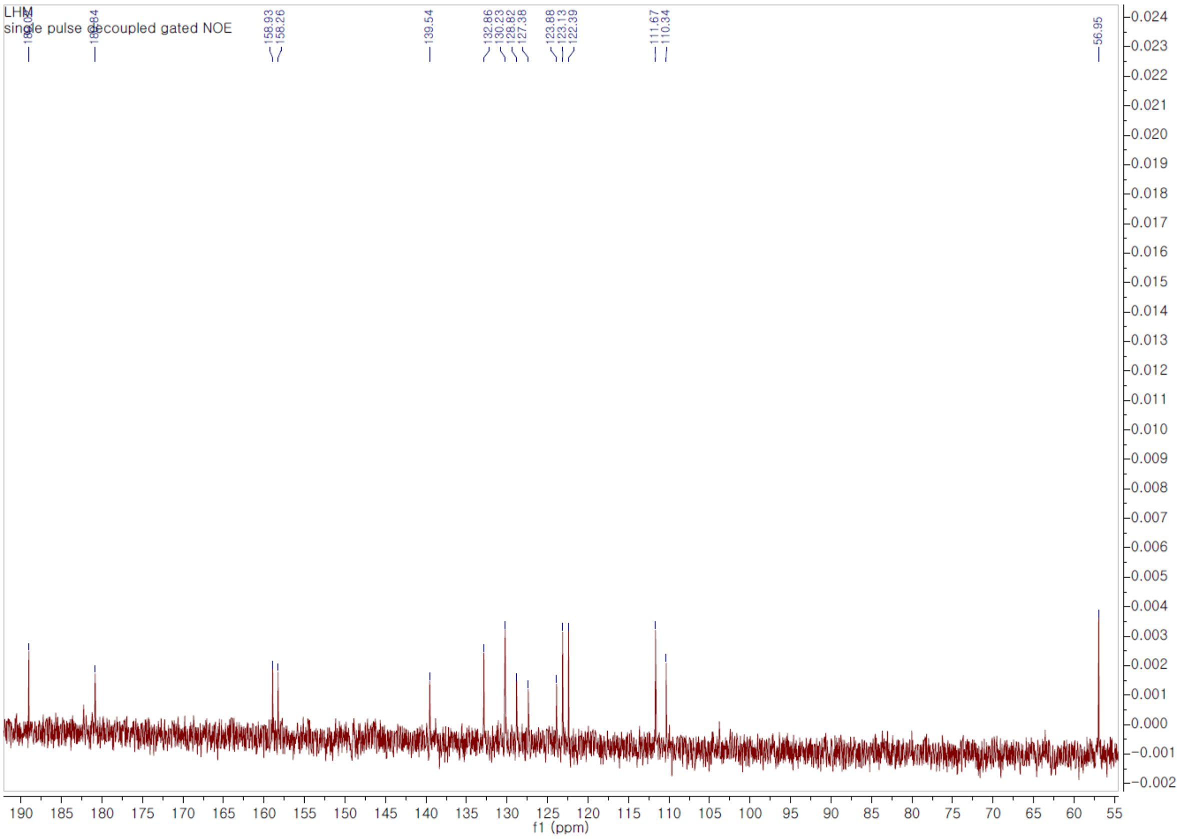

Figure S12. ^{13}C-NMR (125 MHz, DMSO-*d6*) spectrum of compound **3**.

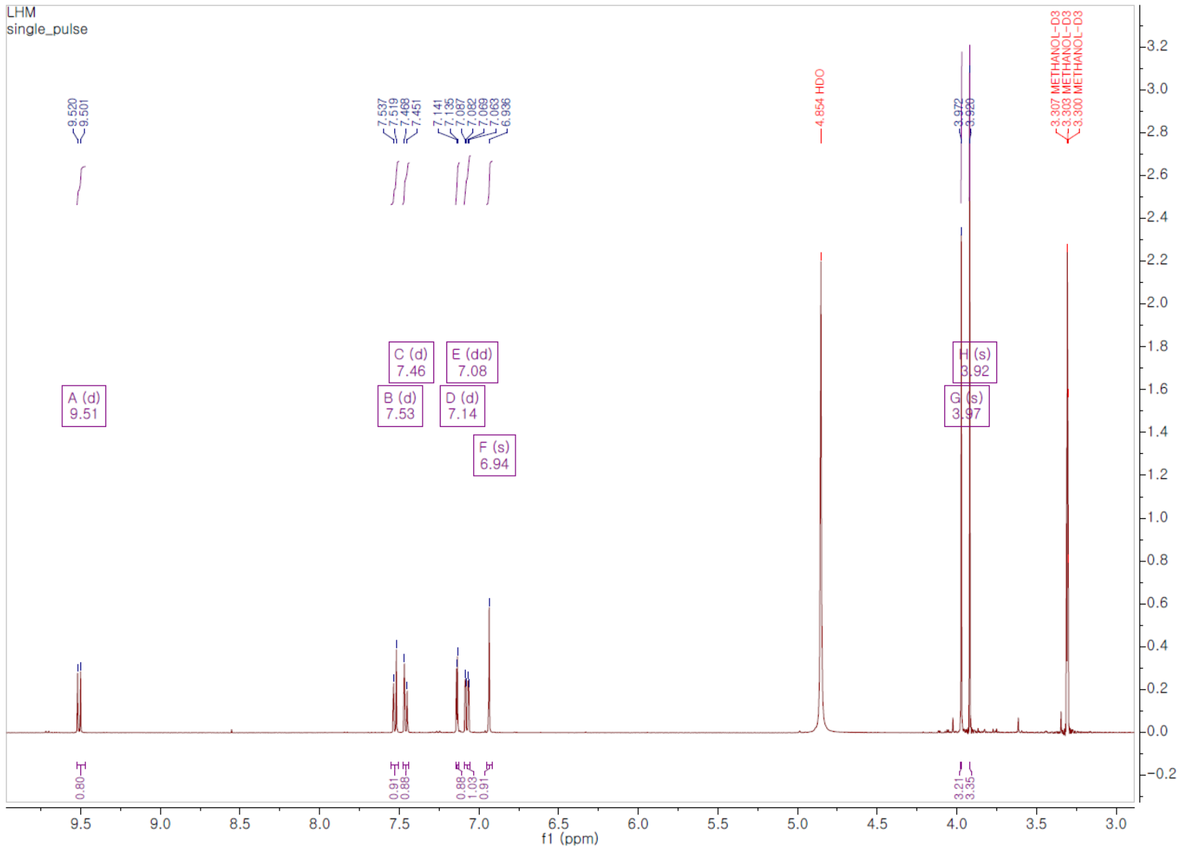

Figure S13. ^{1}H-NMR (500 MHz, CD_3OD) spectrum of compound **4**.

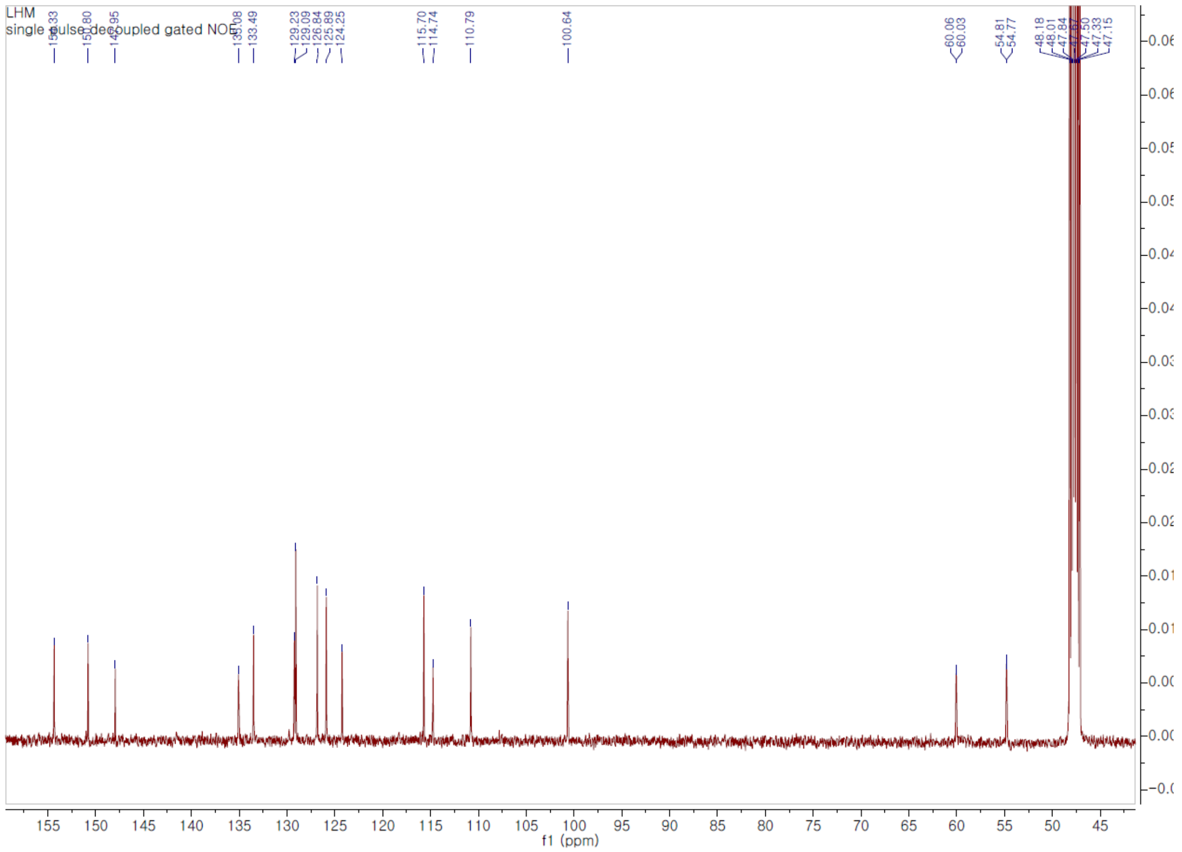

Figure S14. ^{13}C-NMR (125 MHz, CD_3OD) spectrum of compound **4**.

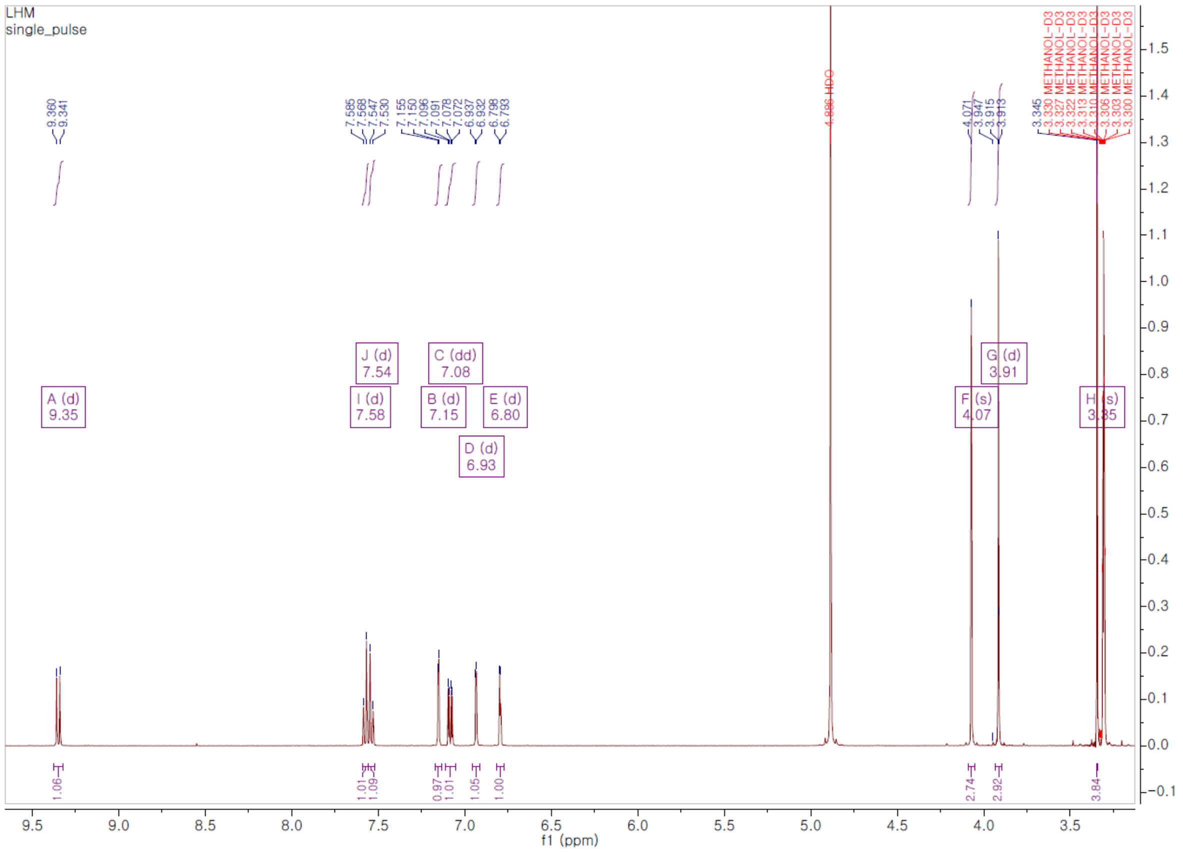

Figure S15. ^{1}H-NMR (500 MHz, CD_3OD) spectrum of compound **5**.

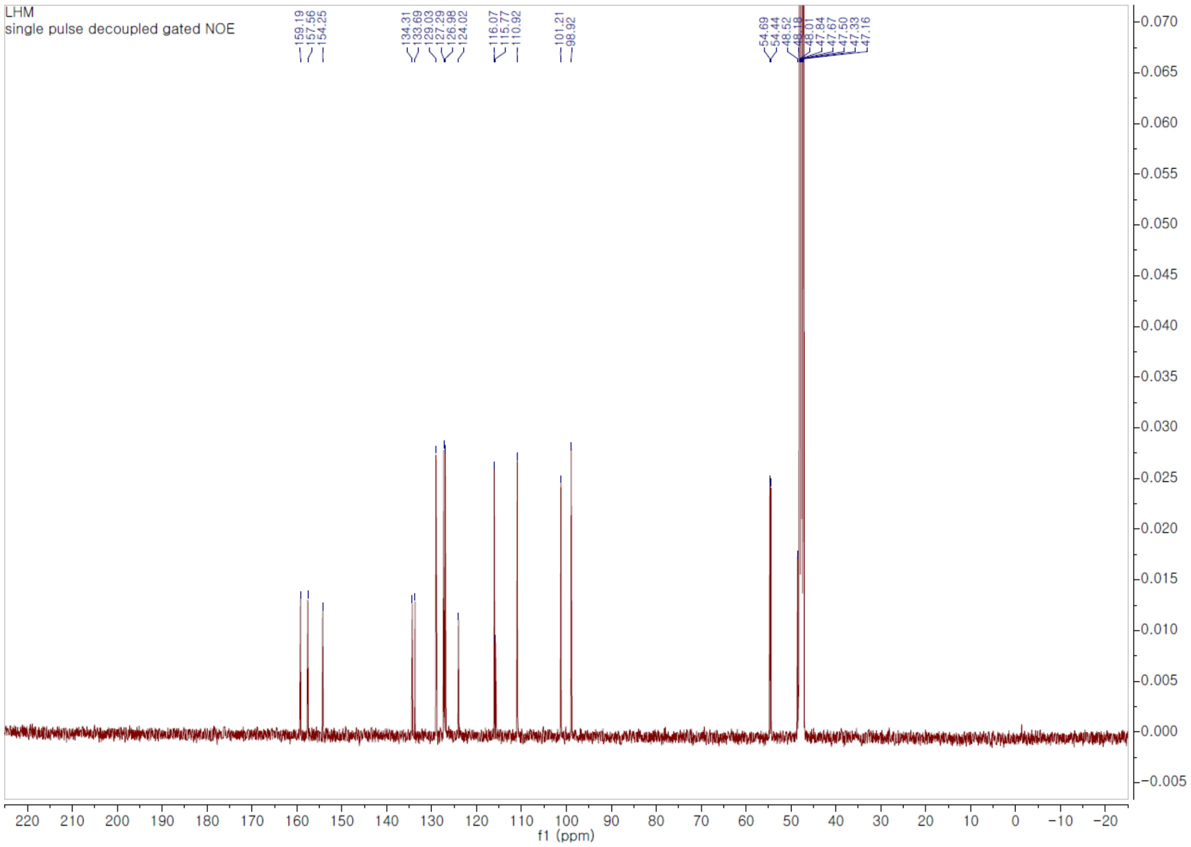

Figure S16. ^{13}C-NMR (125 MHz, CD_3OD) spectrum of compound **5**.

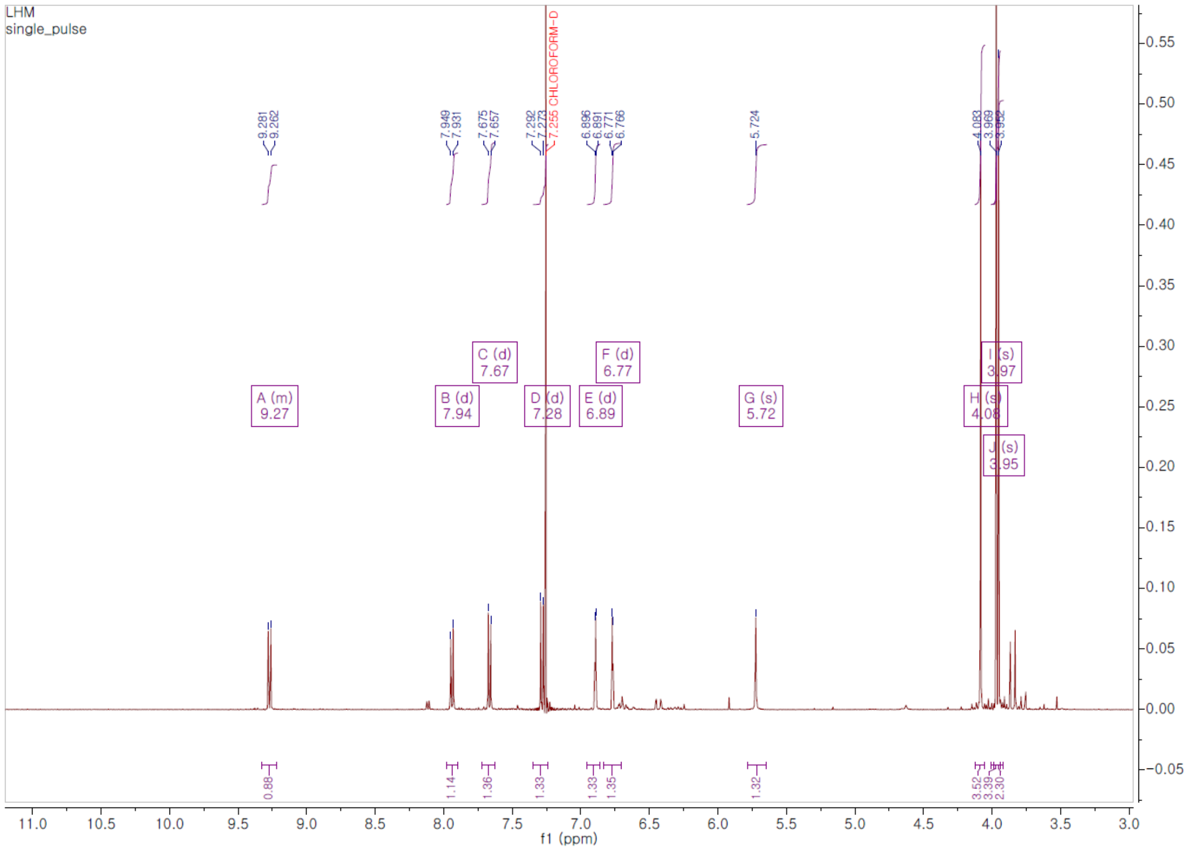

Figure S17. ^{1}H-NMR (500 MHz, $CDCl_3$) spectrum of compound **6**.

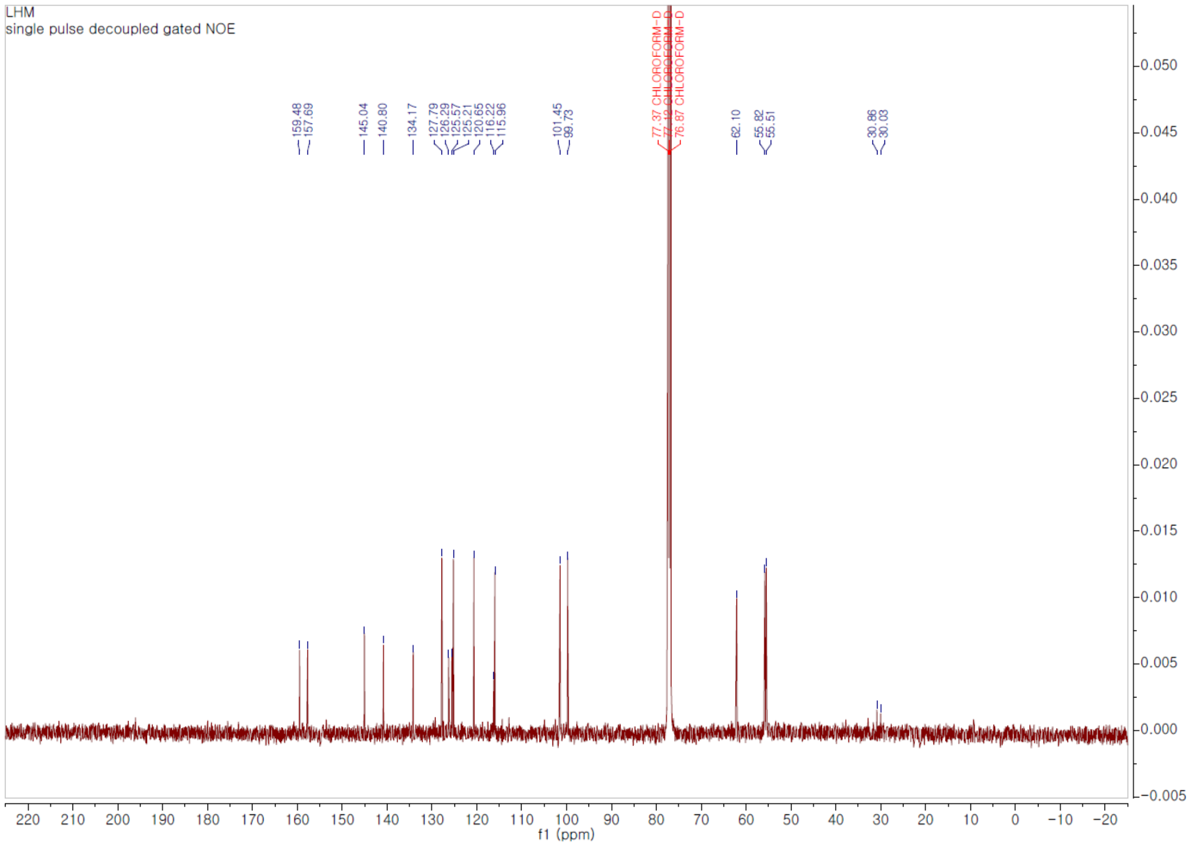

Figure S18. ^{13}C-NMR (125 MHz, $CDCl_3$) spectrum of compound **6**.

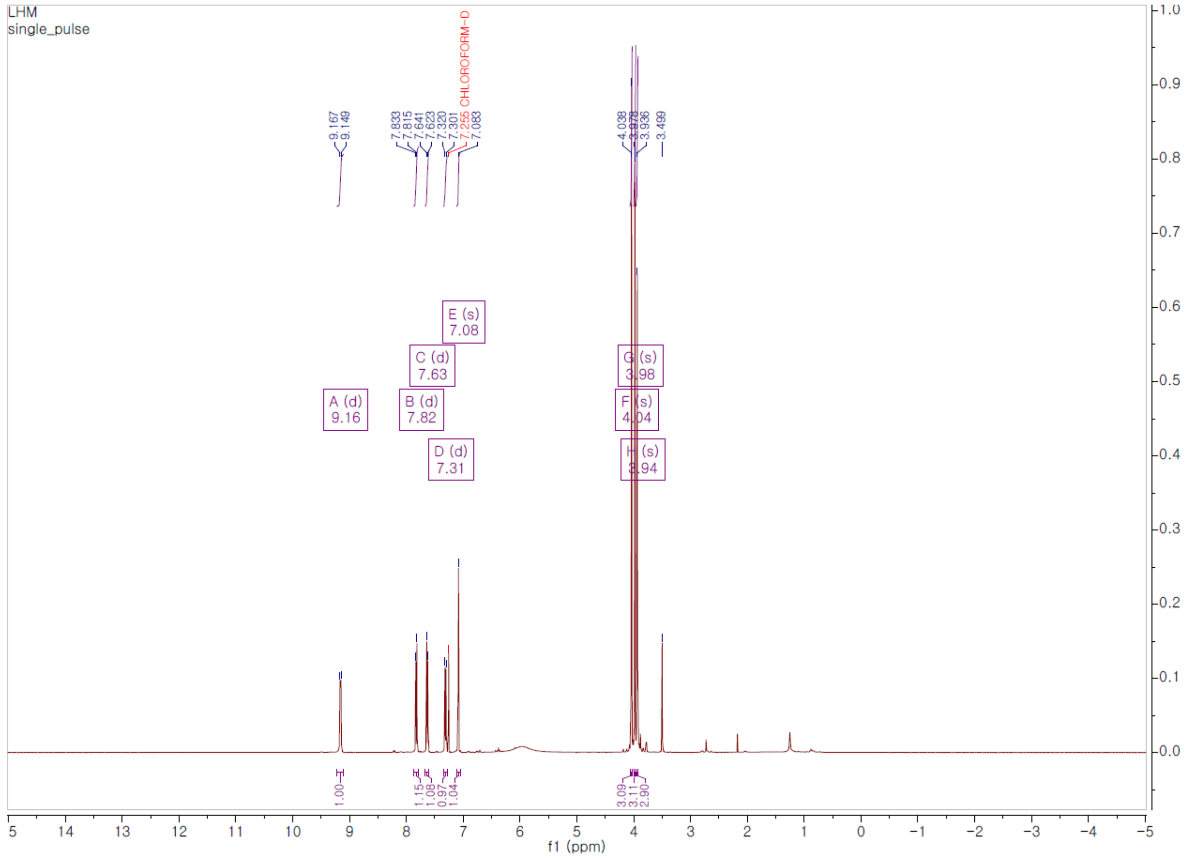

Figure S19. ^{1}H-NMR (500 MHz, $CDCl_3$) spectrum of compound **7**.

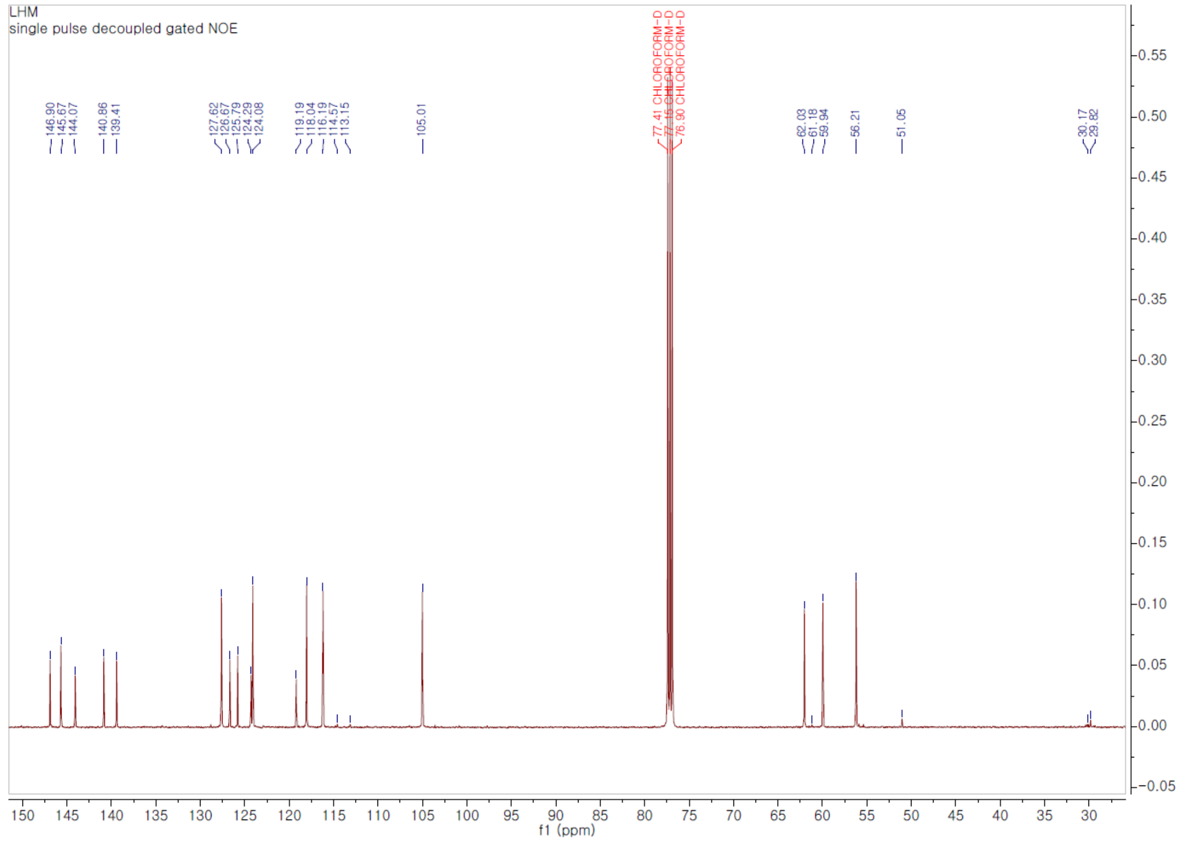

Figure S20. ^{13}C-NMR (125 MHz, $CDCl_3$) spectrum of compound **7**.

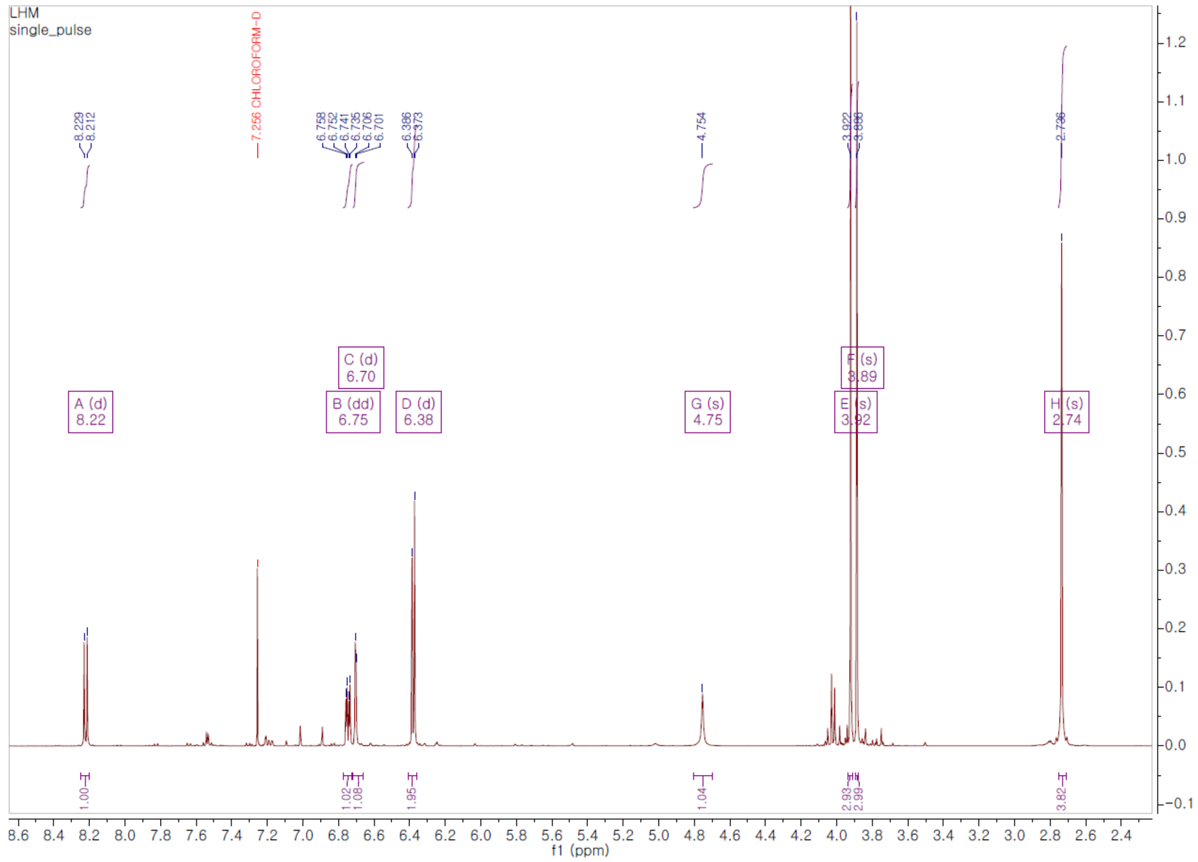

Figure S21. ^{1}H-NMR (500 MHz, $CDCl_3$) spectrum of compound **8**.

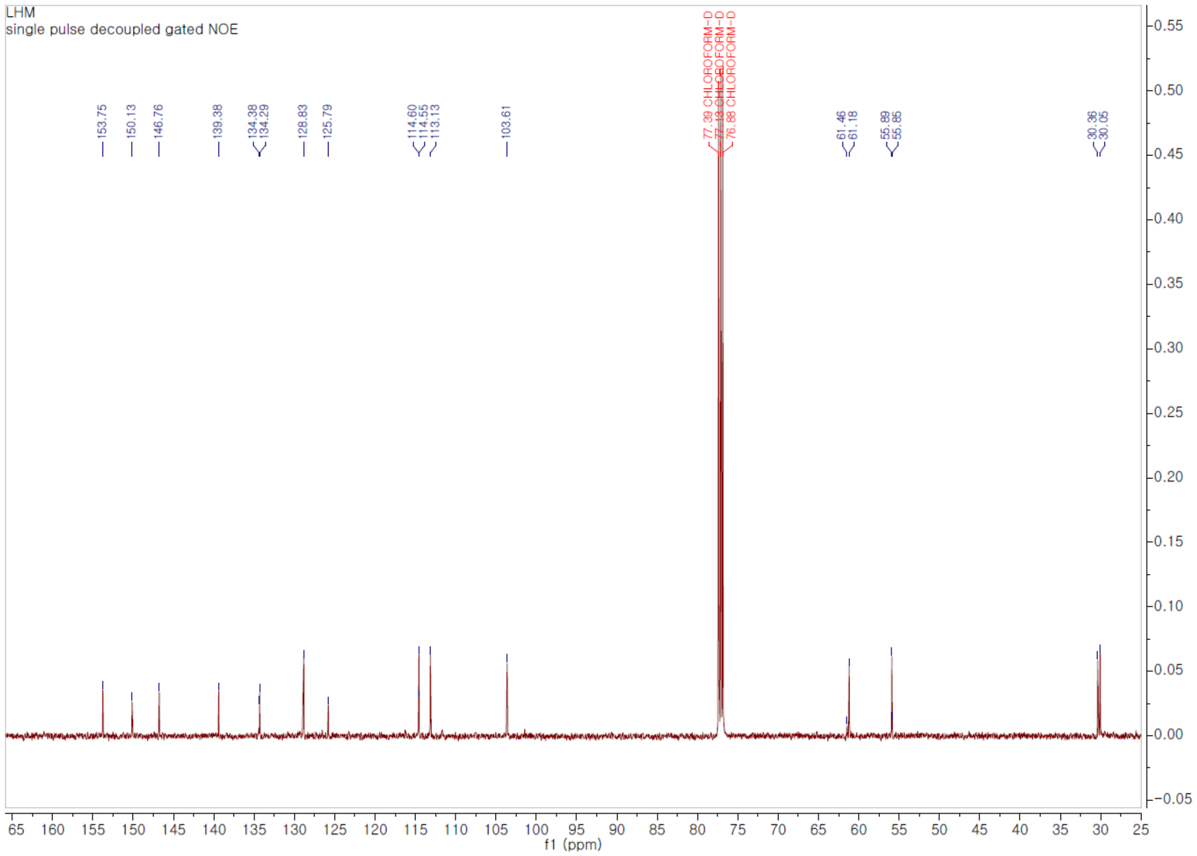

Figure S22. ^{13}C-NMR (125 MHz, $CDCl_3$) spectrum of compound **8**.

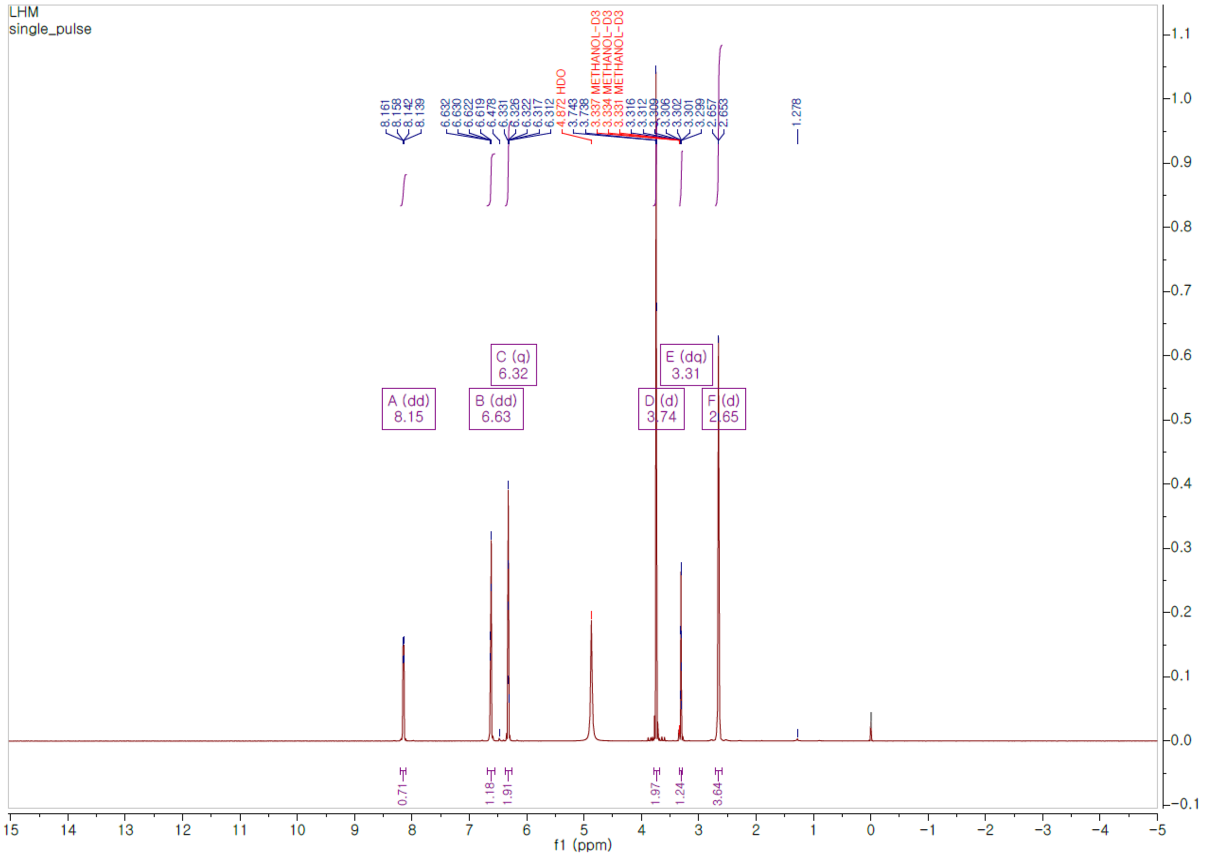

Figure S23. ^{1}H-NMR (500 MHz, CD_3OD) spectrum of compound **9**.

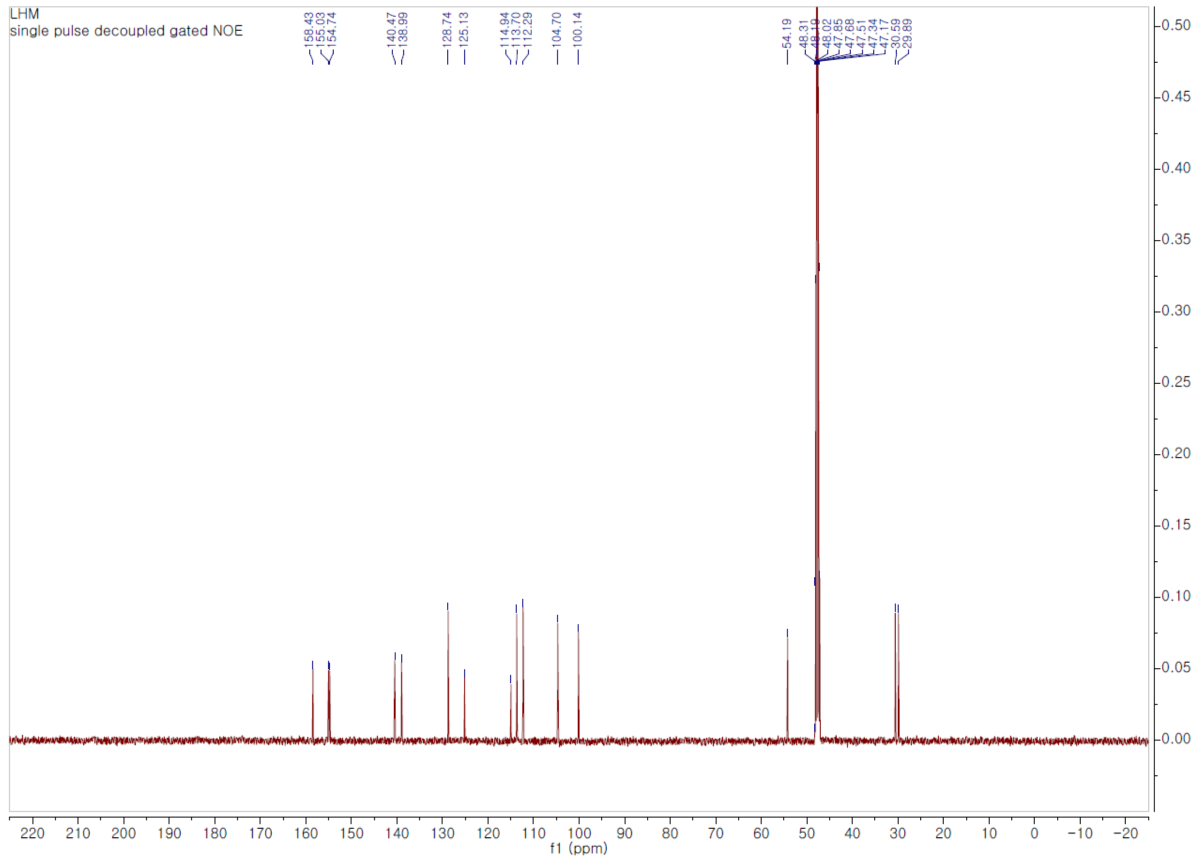

Figure S24. ^{13}C-NMR (125 MHz, CD_3OD) spectrum of compound **9**.

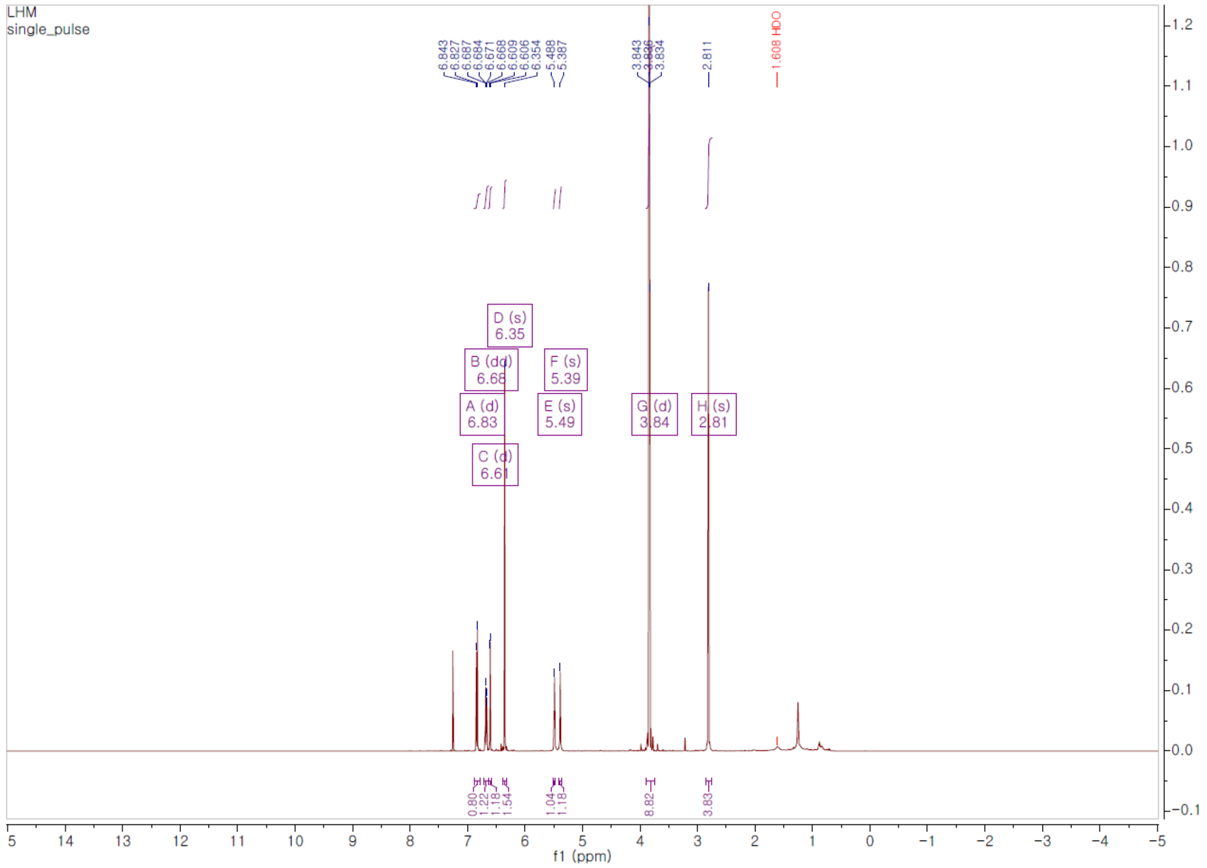

Figure S25. ^{1}H-NMR (500 MHz, $CDCl_3$) spectrum of compound **10**.

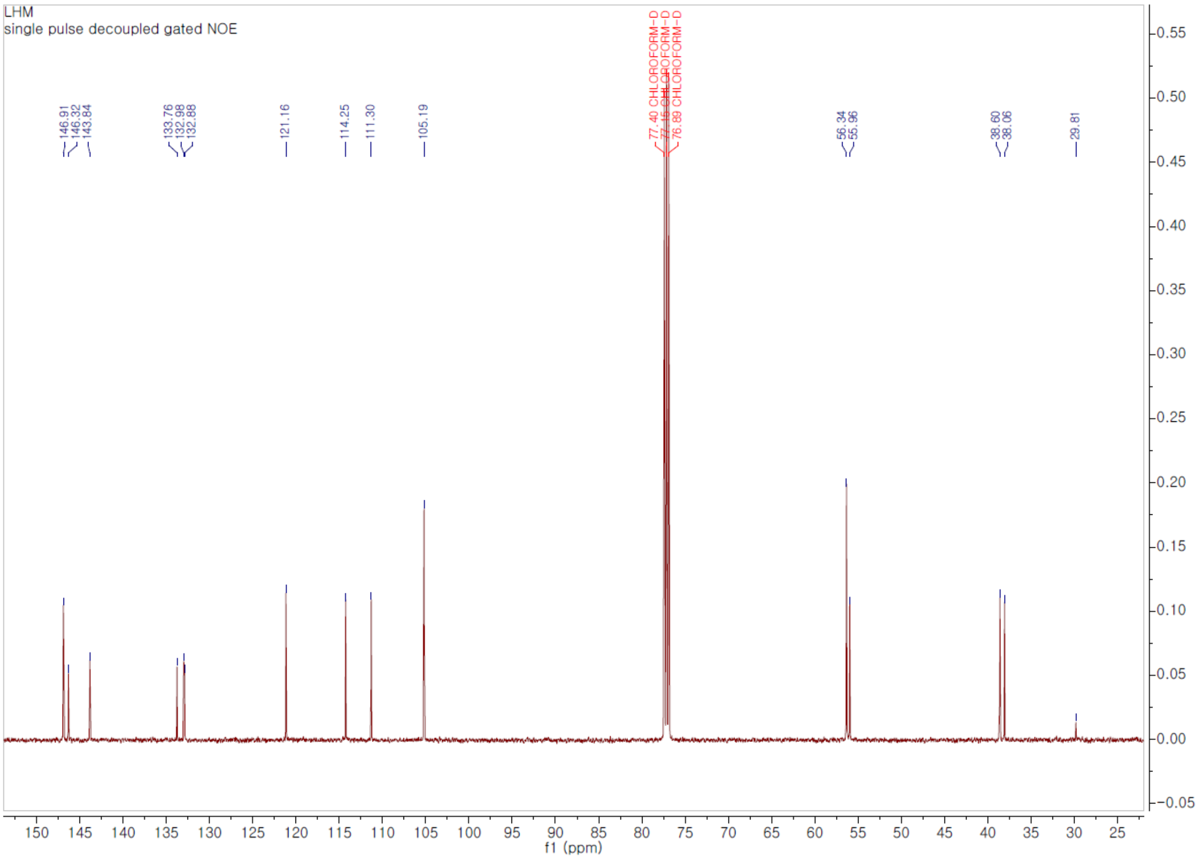

Figure S26. ^{13}C-NMR (125 MHz, CDCl$_3$) spectrum of compound **10**.

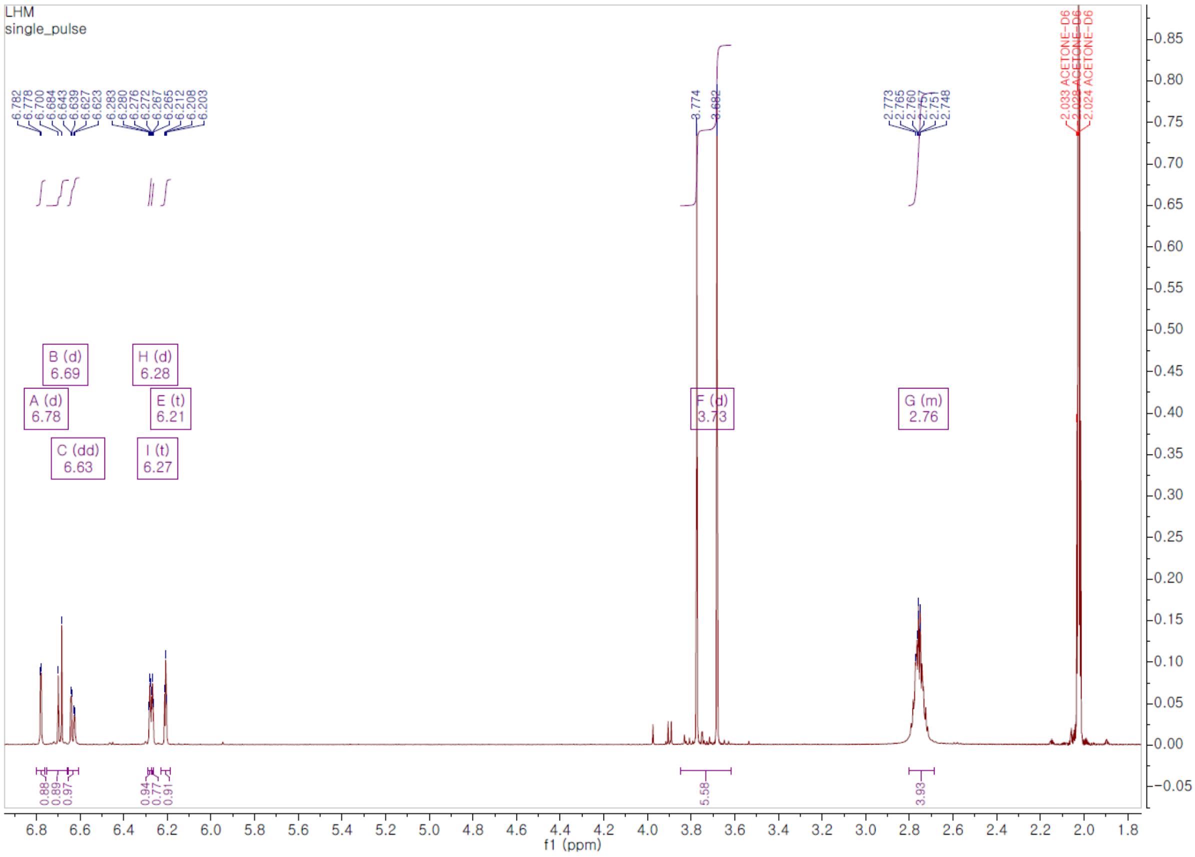

Figure S27. ^{1}H-NMR (500 MHz, acetone-*d6*) spectrum of compound **11**.

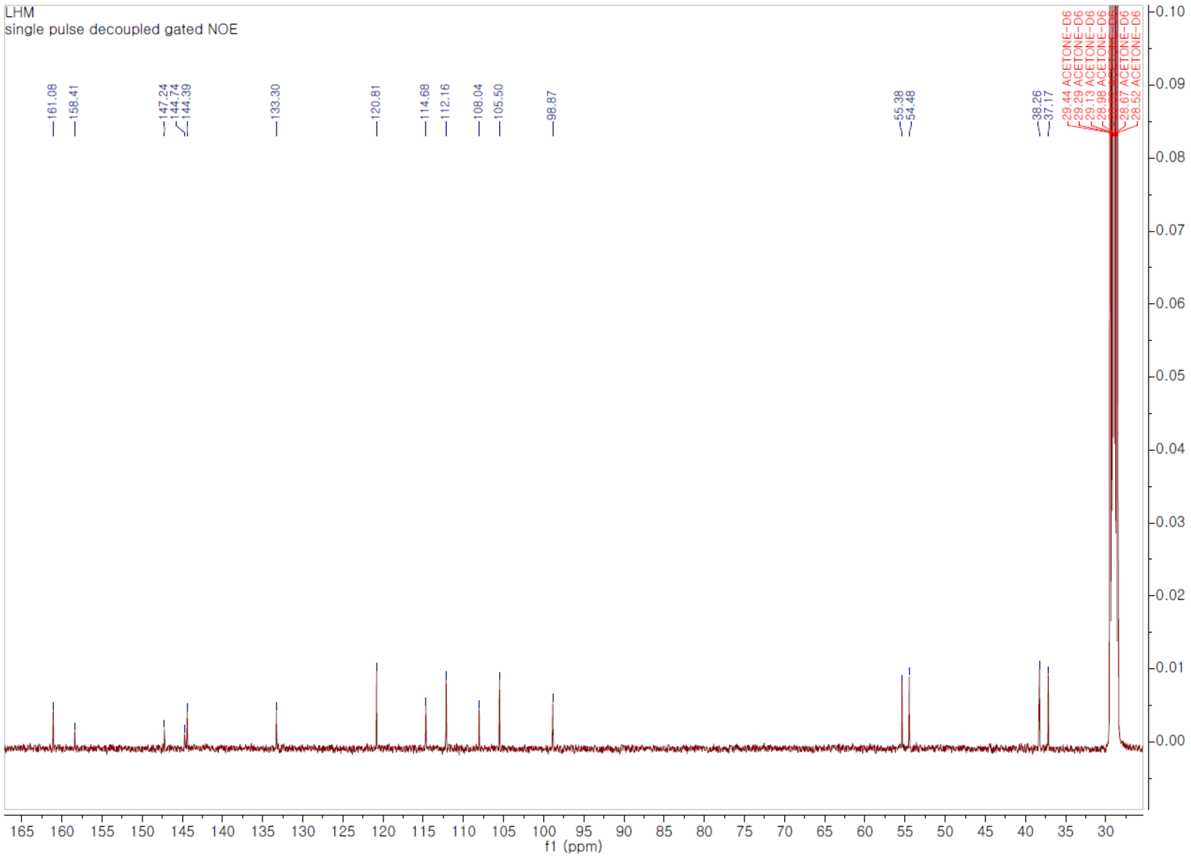

Figure S28. ^{13}C-NMR (125 MHz, acetone-*d6*) spectrum of compound **11**.

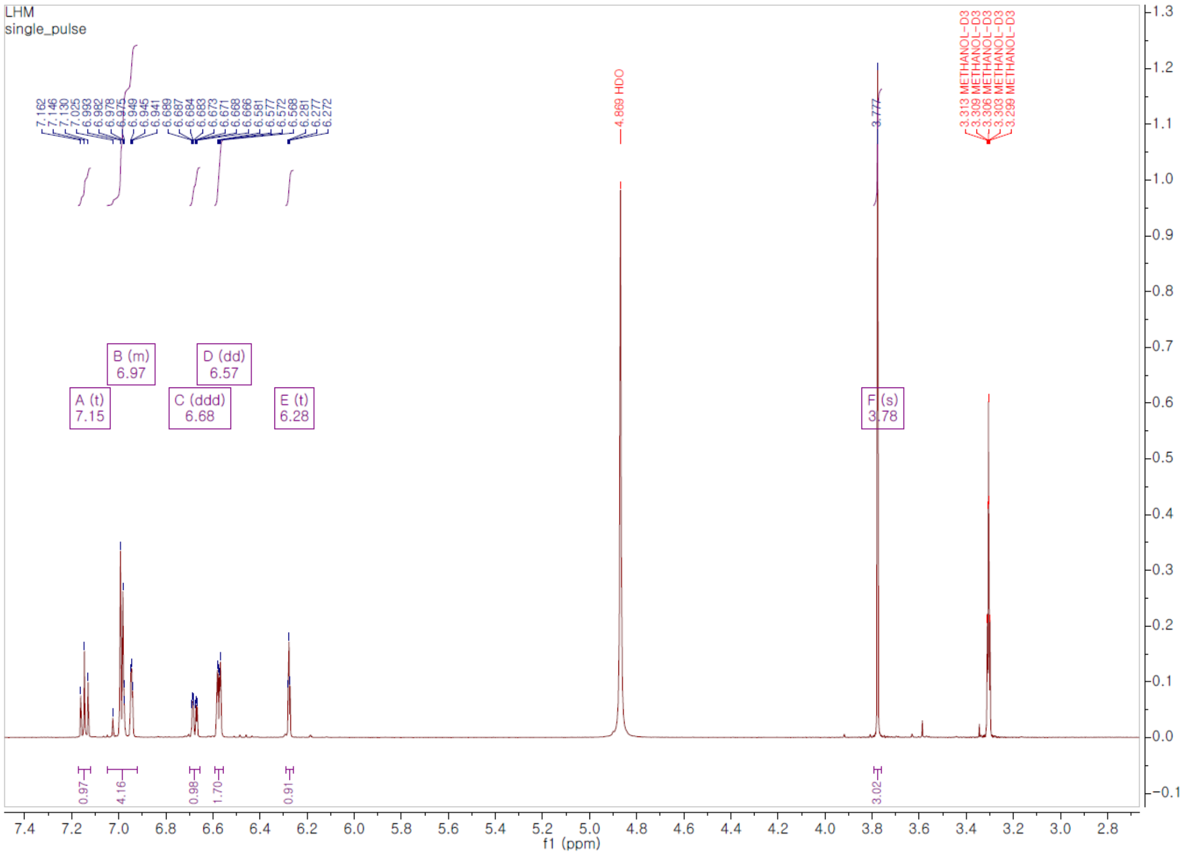

Figure S29. ^{1}H-NMR (500 MHz, CD_3OD) spectrum of compound **12**.

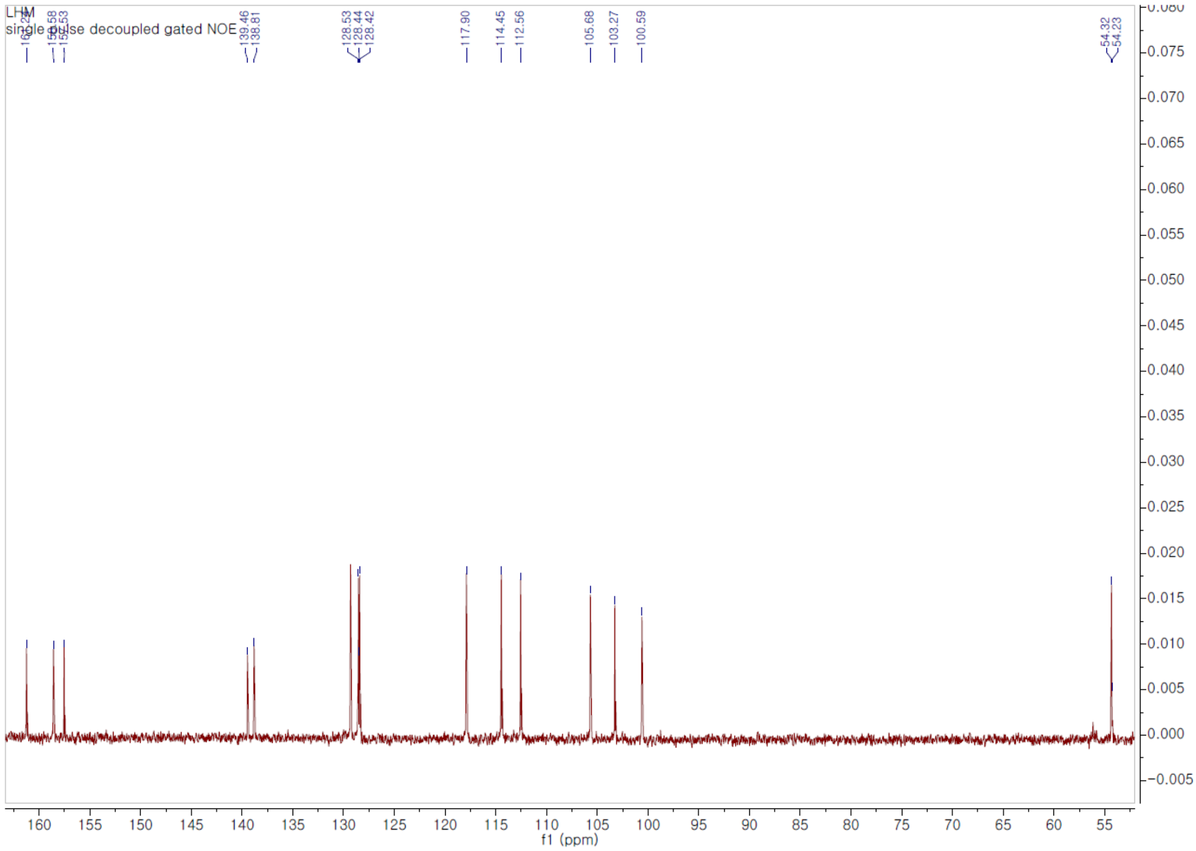

Figure S30. ^{13}C-NMR (125 MHz, CD_3OD) spectrum of compound **12**.

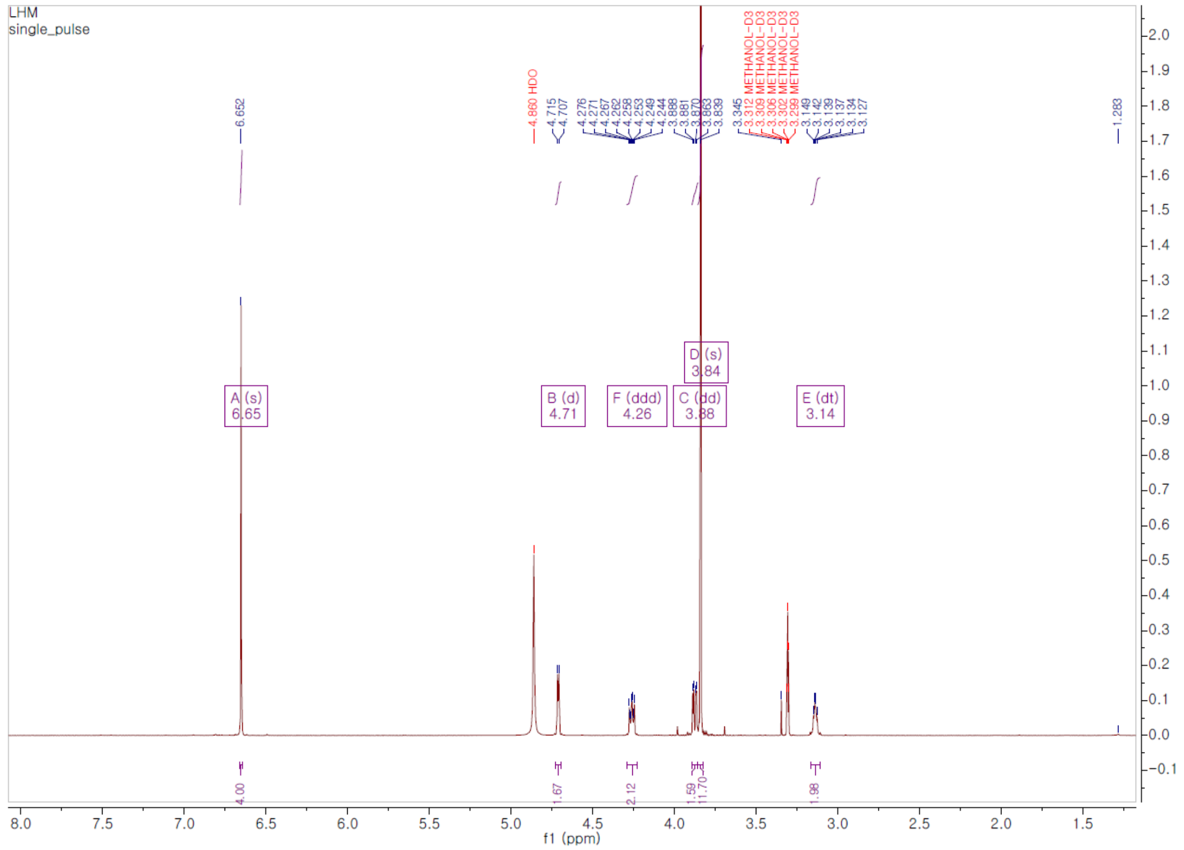

Figure S31. ^{1}H-NMR (500 MHz, CD_3OD) spectrum of compound **13**.

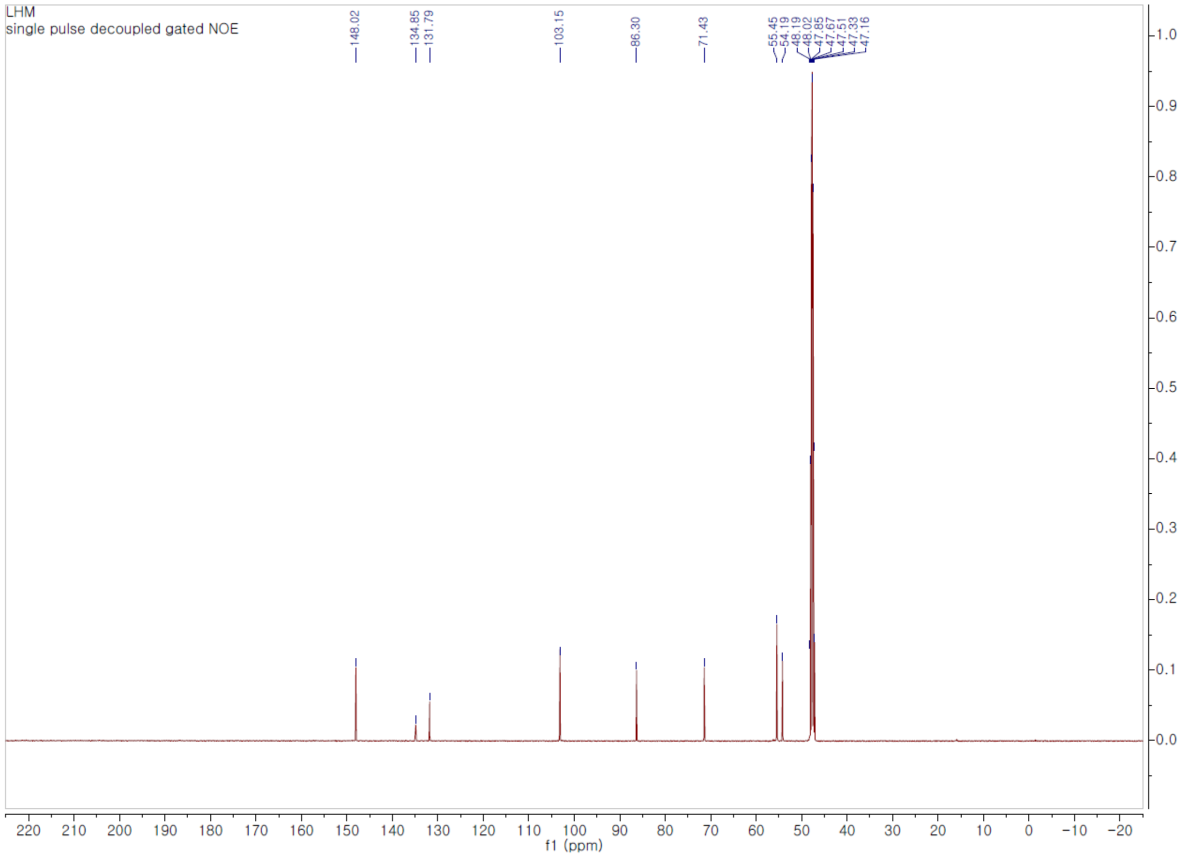

Figure S32. ^{13}C-NMR (125 MHz, CD_3OD) spectrum of compound **13**.

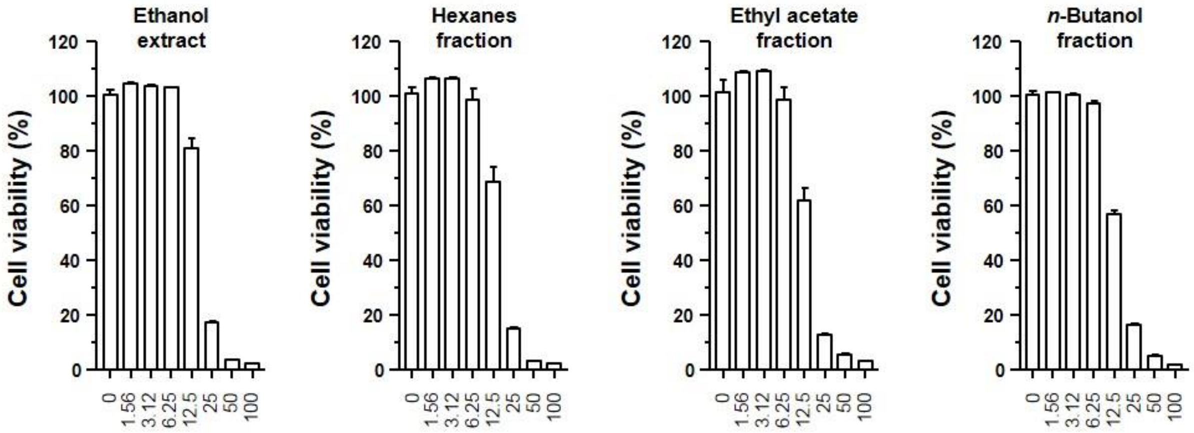

Figure S33. Cytotoxic activities of extract and solvent fractions of Dendrobii Herba against FaDu cell line. Data are presented as means ± SD (n = 6).

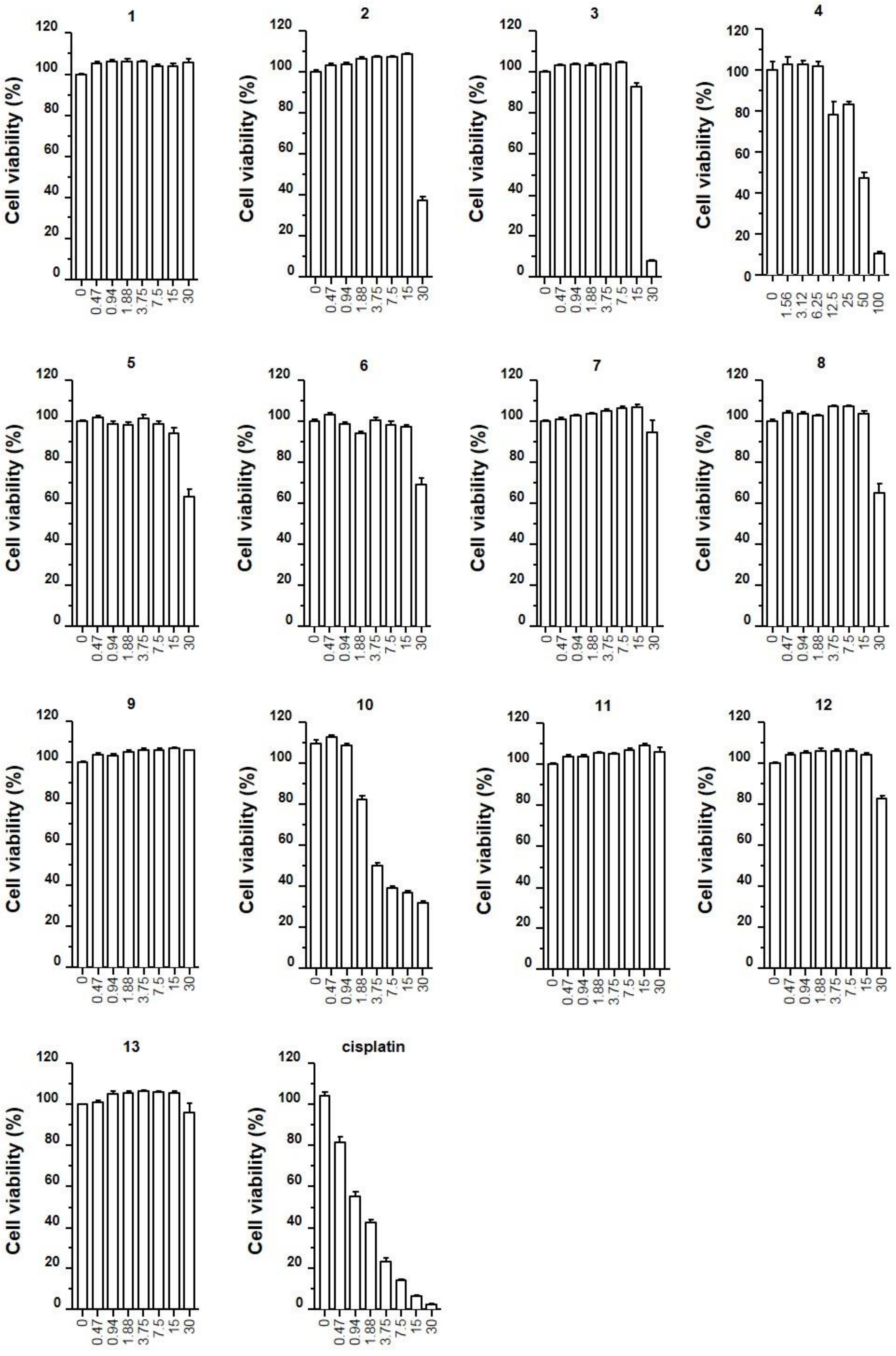

Figure S34. Cytotoxic activities of compounds **1–13** from Dendrobii Herba against FaDu cell line. Data are presented as means ± SD (n = 6).

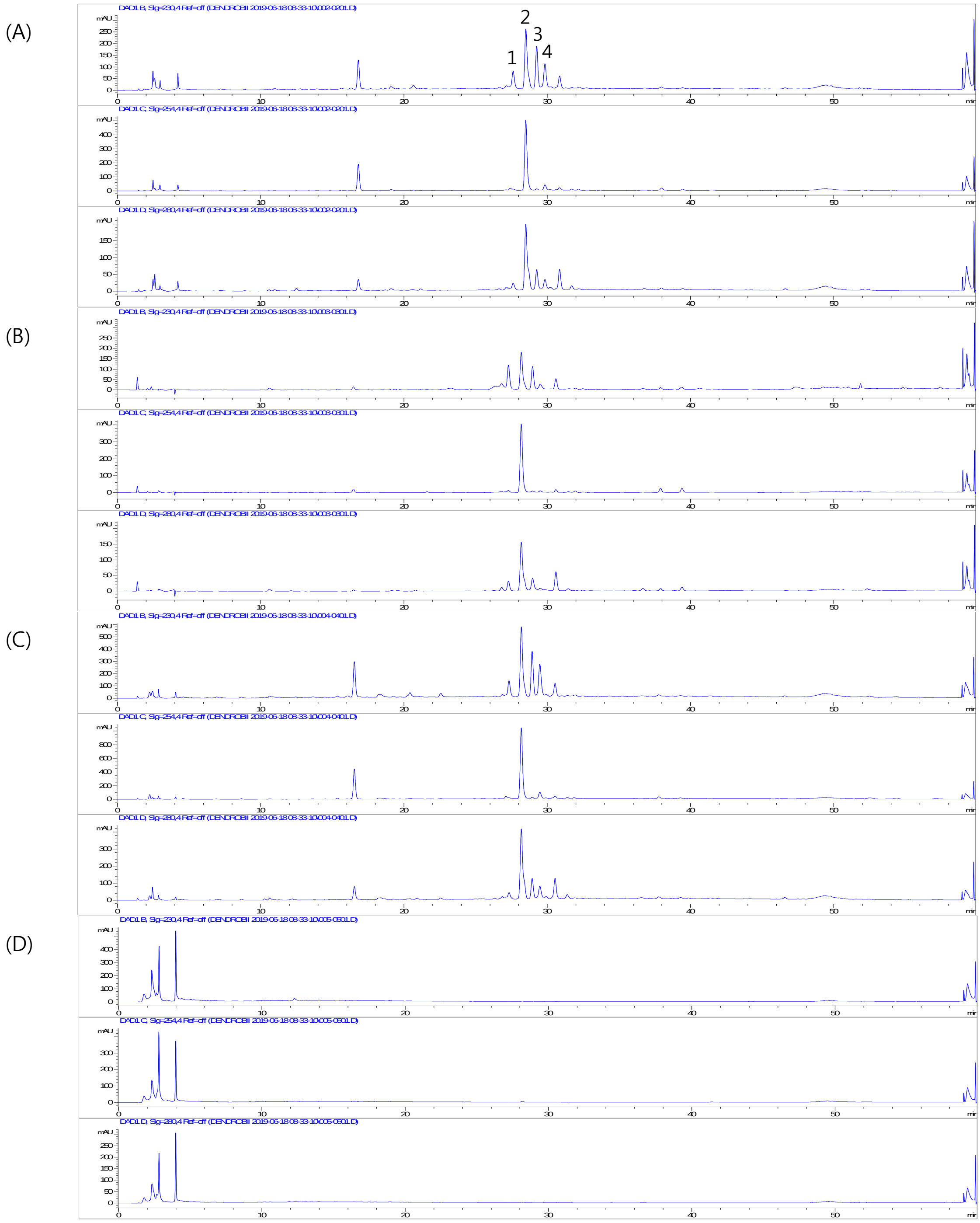

Figure S35. HPLC chromatograms of Dendrobii Herba (A) ethanol extract, (B) hexane soluble fraction, (C) Ethyl acetate soluble fraction and (D) *n*-butanol soluble fraction, detected at 230nm, 254nm and 280nm. Peak Identification by co-injection of each sample with standards: 1, moscatilin; 2, denthyrsinin; 3, gigantol; 4, ephemeranthol A.
